# Regional Environmental Breadth Predicts Geographic Range and Longevity in Fossil Marine Genera

**DOI:** 10.1371/journal.pone.0018946

**Published:** 2011-05-04

**Authors:** Noel A. Heim, Shanan E. Peters

**Affiliations:** Department of Geoscience, University of Wisconsin-Madison, Madison, Wisconsin, United States of America; Zoological Society of London, United Kingdom

## Abstract

**Background:**

Geographic range is a good indicator of extinction susceptibility in fossil marine species and higher taxa. The widely-recognized positive correlation between geographic range and taxonomic duration is typically attributed to either accumulating geographic range with age or an extinction buffering effect, whereby cosmopolitan taxa persist longer because they are reintroduced by dispersal from remote source populations after local extinction. The former hypothesis predicts that all taxa within a region should have equal probabilities of extinction regardless of global distributions while the latter predicts that cosmopolitan genera will have greater survivorship within a region than endemics within the same region. Here we test the assumption that all taxa within a region have equal likelihoods of extinction.

**Methodology/Principal Findings:**

We use North American and European occurrences of marine genera from the Paleobiology Database and the areal extent of marine sedimentary cover in North America to show that endemic and cosmopolitan fossil marine genera have significantly different range-duration relationships and that broad geographic range and longevity are both predicted by regional environmental breadth. Specifically, genera that occur outside of the focal region are significantly longer lived and have larger geographic ranges and environmental breadths within the focal region than do their endemic counterparts, even after controlling for differences in sampling intensity. Analyses of the number of paleoenvironmental zones occupied by endemic and cosmopolitan genera suggest that the number of paleoenvironmental zones occupied is a key factor of geographic range that promotes genus survivorship.

**Conclusions/Significance:**

Wide environmental tolerances within a single region predict both broad geographic range and increased longevity in marine genera over evolutionary time. This result provides a specific driving mechanism for the spatial and temporal distributions of marine genera at regional and global scales and is consistent with the niche-breadth hypothesis operating on macroevolutionary timescales.

## Introduction

Geographic ranges of species and higher taxa vary through space and time in response to environmental perturbations, local biological interactions, and the formation and destruction of physical barriers to dispersal [1]–[4]. Geographic range is an important attribute of taxa because the durations of many marine organisms in the fossil record are positively correlated with geographic range [5]–[13]. Thus, understanding the determinants of geographic range is integral to understanding the nature of extinction dynamics and to building more refined macroevolutionary models. Here we seek to determine if there are fundamental differences in survivorship and environmental breadth between endemic and cosmopolitan marine genera when these attributes are measured within large geographic regions.

One of the first statistical treatments of a large macroecological data set was an analysis of species geographic ranges and ages where John Willis [14] found a strong positive relationship between time since speciation and geographic coverage. This positive relationship lead Willis to propose the so-called age-and-area hypothesis in which species geographic ranges spread as they age. Importantly, Willis explicitly stated that age was not the cause of geographic range, but geographic range expanded as a result of the complex interactions of many factors over time [14]. Because of the importance of time in the age-and-area hypothesis, the fossil record is rich with possibilities for testing this hypothesis. Indeed, the fossil record has upheld the hypothesis that older genera tend to occupy wider geographic ranges [7], [9], [10], [15]. Additionally, Foote et al. [8] found by examining the timing of maximum geographic extent that geographic range expansion promoted survivorship (maximum geographic range early in genus history) just as frequently as age promoted geographic range expansion (maximum geographic range late in genus history). This result corroborates Willis' assertion that geographic range is the time-integrated result of many interacting processes rather than a simple causal relationship. One of the more promising explanations of the age-and-area relationship is the expansion of environmental tolerances. Brown [2] found that living species with large geographic ranges also inhabited a larger variety of niches. In an analysis of the Ordovician radiation Miller [7] found additional support for Brown's hypothesis in that marine genera expanded not only their geographic ranges (measured as the number of paleocontinents occupied) during the Ordovician, but genera also expanded their environmental breadth from onshore to offshore environments. Here we build upon this previous work to test the hypothesis that cosmopolitan marine genera and genera that are endemic to a large continental-scale region experience different macroevolutionary pressures exerted by systematic differences in habitat breadth.

To be clear, there are some important differences between geographic ranges and habitat occupancy observed for living taxa and those observed for fossil taxa. First, many studies of the geographic ranges of living taxa seek to understand spatial patterns of abundance (e.g., [2]–[4], [16]–[18]). Although abundance information is typically preserved in fossil assemblages [19], abundance information and sampling density within range bounds is typically not available for large macroecological studies. Consequently, geographic ranges of fossil taxa discussed here are measures of the extent of occurrence, not the area of occupancy within the range [20]. Furthermore, observed geographic ranges of living taxa are compiled over relatively short intervals of time whereas fossil ranges are averaged over geologic time scales (typically a few million years). However, the advantage of studying time-averaged geographic ranges is that high frequency variations (e.g., seasonal, decadal) in areal extent are averaged out [21]. The time dimension of the fossil record, of course, allows geographic ranges to be incorporated into macroevolutionary theory (e.g., [1], [5]–[7], [10], [13], [15], [22]).

There should be a positive relationship between the areal extent of a taxon and the number of distinct environments encompassed by the overall geographic range during any given geological instant. This relationship holds because the surface of the Earth, including the continental shelves, is spatially heterogeneous with respect to soil/substrate type, temperature, seasonality, angle of incidence for insolation, nutrient availability, etc. [2]. This makes good intuitive sense and at large spatial scales it must be true. However, just because habitats become increasingly heterogeneous at larger spatial scales, taxa do not actually need to exploit a greater number of habitats as spatial scale increases. Gaston [4], for example, argues that taxa with large geographic ranges do not necessarily occupy more habitat-types than more narrowly ranging taxa, but rather they occupy more widely distributed habitats. Furthermore, sampling bias has been shown to explain nearly all the covariation between range size and environmental tolerance [23]. However, only broad marine habitat types based on bathymetry, substrate-type and paleo-oxygenation can be identified in the marine stratigraphic record; identifying specific resources that limit taxa (e.g., [23]) is also exceedingly difficult on the basis of fossils. Marine habitats are also frequently time-transgressive, which means that they move through space during long intervals of sea level change. As a consequence, the observed geographic range of a taxon in any given geologic time interval is likely to be larger than it was during any instant in time. Geographic ranges observed in the fossil record are, therefore, time integrated, and taxa with large ranges may reflect a strong tendency towards habitat tracking [24] rather than wide environmental tolerances. Here we test the hypothesis that globally distributed taxa have different macroevolutionary responses to environmental change by comparing genus duration, per-interval geographic range size, and paleoenvironmental occupancy.

**Figure 1:**
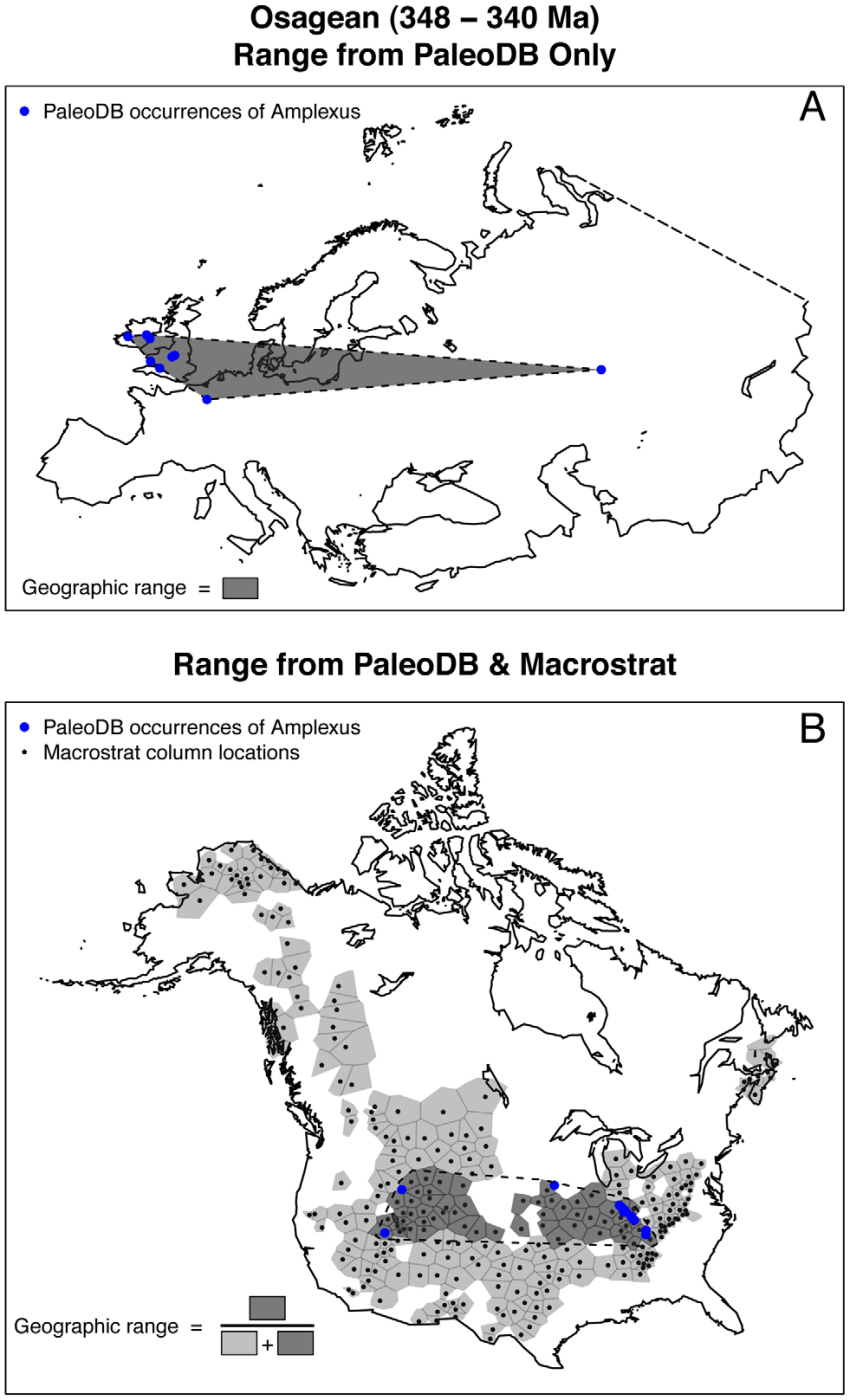
Maps of Europe and North America illustrating the two geographic range calculation methods. The large blue dots indicate the position of Osagean (early Mississippian) PaleoDB occurrences of the rugose coral genus *Amplexus*. (A) Map of Europe, including all of Turkey, showing the convex hull method of calculating geographic range. The blue points show the location of Osagean aged PaleoDB collections containing *Amplexus*. The geographic range of *Amplexus* is the area within the convex hull around all PaleoDB collections. The far eastern point is an outlier, but not an error. There are other occurrences of *Amplexus* in eastern Europe during other stages of the Mississippian. This method of geographic range calculation was also applied to all North America genera. (B) Map of North America illustrating the Macrostrat method of calculating geographic range. The small black points and shaded polygons represent locations and areas of stratigraphic summary columns in Macrostrat [47] that have Osagean age marine sedimentary rocks. The dark polygons are those whose column location is inside the convex hull (dashed line) drawn around the set of Macrostrat columns that are closest to each of the PaleoDB collections. The total geographic range of *Amplexus* during the Osagean is the total area of dark polygons and the proportional geographic range is the ratio of dark to all polygons.

We compiled from the Paleobiology Database (http://paleodb.org) information on 3303 endemic and 2716 cosmopolitan marine genera from North America and 3788 endemic and 3360 cosmopolitan marine genera from Europe. Geographic ranges were calculated on a per interval basis as the convex hull around PaleoDB occurrences of each genus (Fig. 1A) in North America and Europe. We also took advantage of the Macrostrat database (http://macrostrat.org), which currently does not include Europe, to quantify broad scale changes in the sedimentary cover of North America and to employ an alternative measurement of geographic range (Fig. 1B). The spatial structure of Macrostrat permits geographic ranges to be measured as the proportion of preserved marine sediments, which is a reflection of the area of shallow marine shelves that is occupied by a given genus (Fig. 1B; see Materials and Methods). This methodology is useful because it allows geographic range to be measured relative to a time-varying quantity: the total area of preserved marine shelf environments. We define an endemic genus as one whose observed geographic range is confined to North America or Europe for its entire duration. Genera with contemporaneous occurrences within and outside of the focal region (Europe or North America) are considered cosmopolitan (see Materials and Methods).

Our definition of endemism (genera with fossil occurrences contained within the political boundaries of Europe or the United States and Canada) is free from *a priori* interpretations of the paleontologic and paleoenvironmental properties of the region [25]. Our analyses are also restricted to genera, which have geographic ranges and durations that reflect the combined properties of their constituent species. Because macroevolutionary processes do not always propagate uniformly up the taxonomic hierarchy [26], the union of those processes that determine geographic ranges in species and genera may not equal their intersection. For example, there is evidence based on living marine bivalves [22] and large Pleistocene mammals [27] that many species within widespread genera have largely overlapping geographic ranges or occupy the same biogeographic province and that only a small number of the constituent species exist outside the “typical” range for species of the genus. In other words, the species within a genus are not necessarily distributed uniformly within the overall geographic range. Furthermore, because most compilations of Phanerozoic marine diversity are made at the genus-level or family-level (e.g., [28]–[30]), understanding the dynamics of genus-level biogeographic processes are important for interpreting Phanerozoic diversity patterns.

We interpret the age-and-area relationship in terms of environmental breadth, as measured by the number of paleoenvironmental zones occupied [7] by genera both on a per-interval basis and across the lifetime of each genus. We also measure rates of environmental turnover for North America using Macrostrat and the principals of macrostratigraphy [31]. Previous results from Macrostrat have demonstrated that times of reduced marine sedimentation on the North America craton correspond to times of increased extinction in marine genera [32], but we now use this approach to test the hypothesis that endemic genera have higher extinction susceptibility in the face of large-scale environmental reorganizations than cosmopolitan genera.

## Results

We present here the results of analyses of genus duration and geographic range for two continental-scale data sets: Europe and North America. We analyzed geographic range of North American genera using both the traditional convex hull method based only on PaleoDB collection locations and using the area of available rock record in Macrostrat. Geographic ranges for Europe were only tabulated using a convex hull around PaleoDB collection locations. Results for all three combinations of data sets and geographic range calculation methods are presented, but note that there are few differences between Europe and North America and that there are no systematic differences within North America for the two methods of geographic range estimation.

As with any sampled range data, both stratigraphic duration and geographic range are sensitive to sampling intensity. However, stratigraphic duration is not only sensitive to the number of samples, it's also sensitive to true (not sampled) geographic range. Even if true duration is the same for all taxa, those with smaller geographic ranges will appear to have shorter observed durations than genera that are widespread, unless stratigraphic and spatial sampling is dense [33]. The paleontological data in the PaleoDB is not sufficiently dense to overcome this type of sampling bias, but sampling is not an insurmountable problem. First, we are not comparing widespread taxa to narrowly distributed taxa; we are assessing whether or not two groups of taxa have different spatial and temporal distributions. Furthermore, we recognize that sampling intensity will bias results and therefore we attempt to correct for sampling by comparing only those groups of taxa with similar sampling intensities and by partial correlation, wherein sampling intensity is factored out.

**Figure 2:**
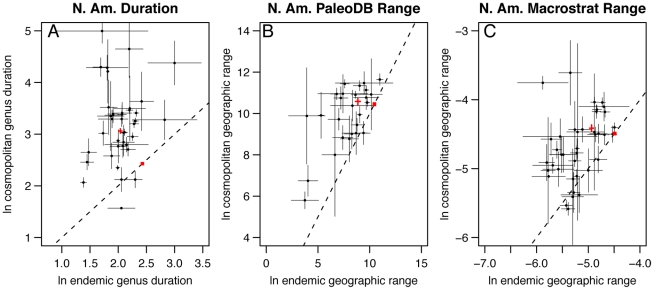
Mean genus duration and geographic range for 39 Linnaean classes in North America. The red crosses are ± two standard errors around the mean for all genera in each category. The red boxes outline the expected values for all endemic and cosmopolitan genera if durations and geographic ranges were randomly distributed among taxa. The boxes show the middle 95% of values from 10,000 bootstrapping iterations. Note the significant disjunct between observed and expected mean genus durations and geographic ranges. (A) Endemic vs. cosmopolitan duration within North America. (B) Endemic vs. cosmopolitan geographic range, calculated as a convex hull around PaleoDB collections, within North America. (C) Endemic vs. cosmopolitan geographic range, calculated as proportion of the total available rock area occupied by a genus within North America. Only one value for each genus (the mean geographic range) is used in the per class calculations. Error bars are ± one standard error of class mean. The one-to-one line (dashed) is plotted for reference. A key to class identity along with observed and expected values for the aggregation of all genera are given in Figure S2.

**Figure 3:**
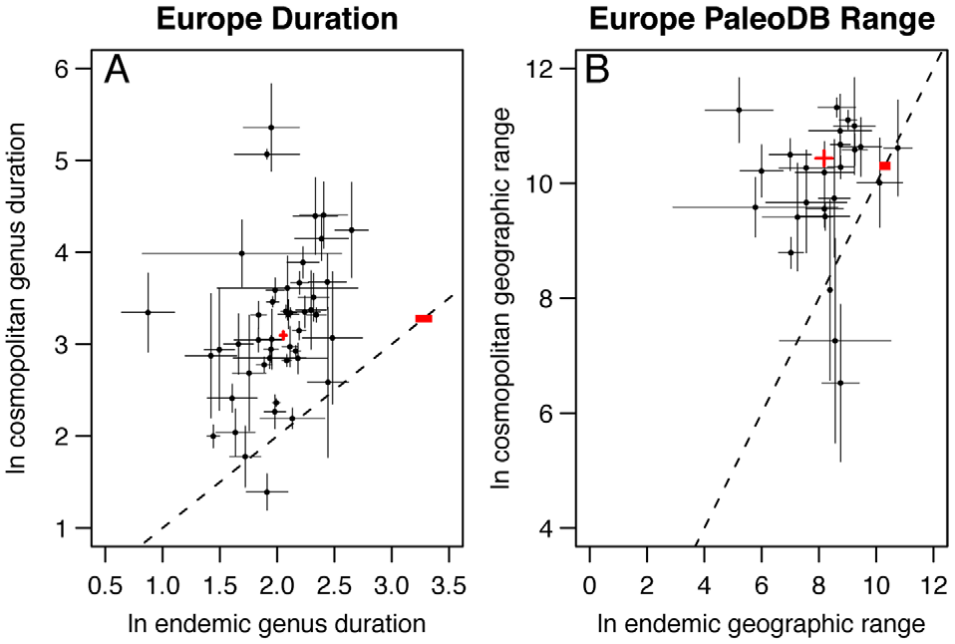
Mean genus duration and geographic range for 39 Linnaean classes in Europe. The red crosses are ± two standard errors around the mean for all genera in each category. The red boxes outline the expected values for all endemic and cosmopolitan genera if durations and geographic ranges were randomly distributed among taxa. The boxes show the middle 95% of values from 10,000 bootstrapping iterations. Note the significant disjunct between observed and expected mean genus durations and geographic ranges. (A) Endemic vs. cosmopolitan duration within Europe. (B) Endemic vs. cosmopolitan geographic range, calculated as a convex hull around PaleoDB collections, within Europe. Only one value for each genus (the mean geographic range) is used in the per class calculations. Error bars are ± one standard error of class mean. The one-to-one line (dashed) is plotted for reference. A key to class identity along with observed and expected values for the aggregation of all genera are given in Figure S2.

**Figure 4:**
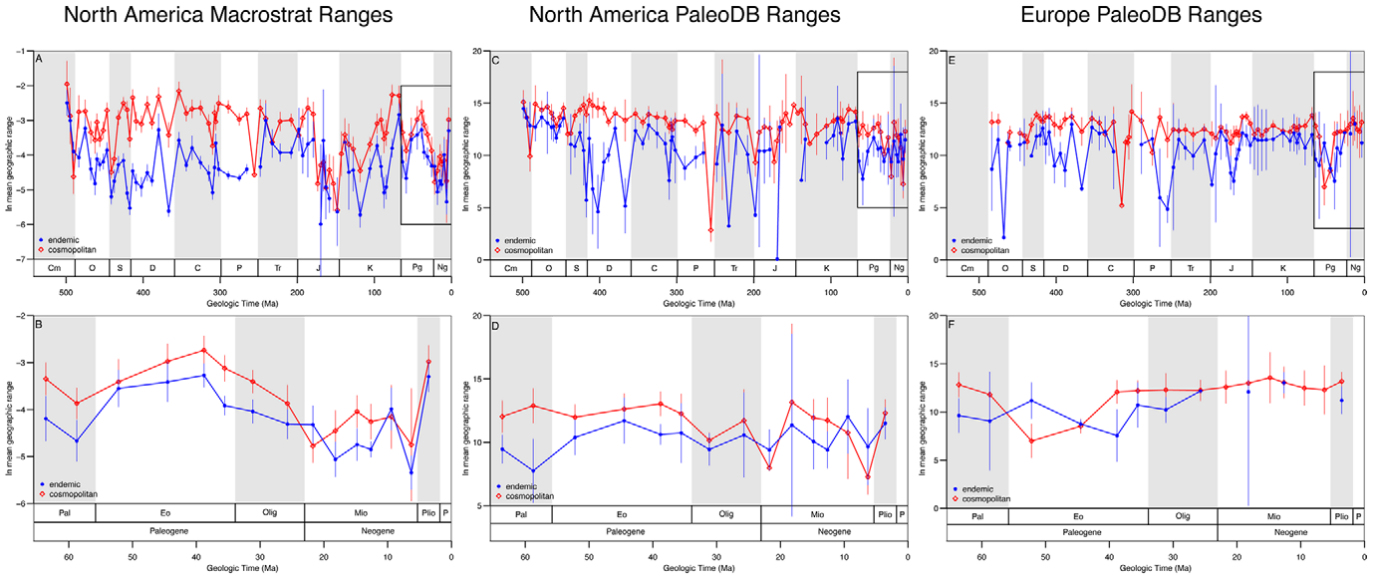
Mean geographic ranges of endemic genera and cosmopolitan genera within North America and Europe. Error bars are ± two standard errors of the mean. (A) The Phanerozoic time series of mean geographic range in North America tabulated using Macrostrat. Breaks in the endemic data at the late Permian and late Jurassic indicate times for which there are no marine genera endemic to North America in the Paleobiology Database. The box shows the portion of the time scale expanded in the plot below. Points are plotted at the geologic time interval midpoints. (B) An expansion of the Cenozoic portion of the time series is presented in the plot above. (C) The Phanerozoic time series of mean geographic range in North America was tabulated using the simple convex hull method of calculating geographic range. (D) An expansion of the Cenozoic portion of the time series is presented in the plot above. (E) The Phanerozoic time series of mean geographic range in Europe was tabulated using the simple convex hull method of calculating geographic range. (F) An expansion of the Cenozoic portion of the time series is presented in the plot above. The Phanerozoic time scale abbreviations are as follows: Cm, Cambrian; O, Ordovician; S, Silurian; D, Devonian; C, Carboniferous; P, Permian; Tr, Triassic; J, Jurassic; K, Cretaceous; Pg, Paleogene; Ng, Neogene. The Cenozoic time scale abbreviations are as follows: Pal, Paleocene; Eo, Eocene; Olig, Oligocene; Mio, Miocene; Plio, Pliocene; P, Pleistocene.

**Figure 5:**
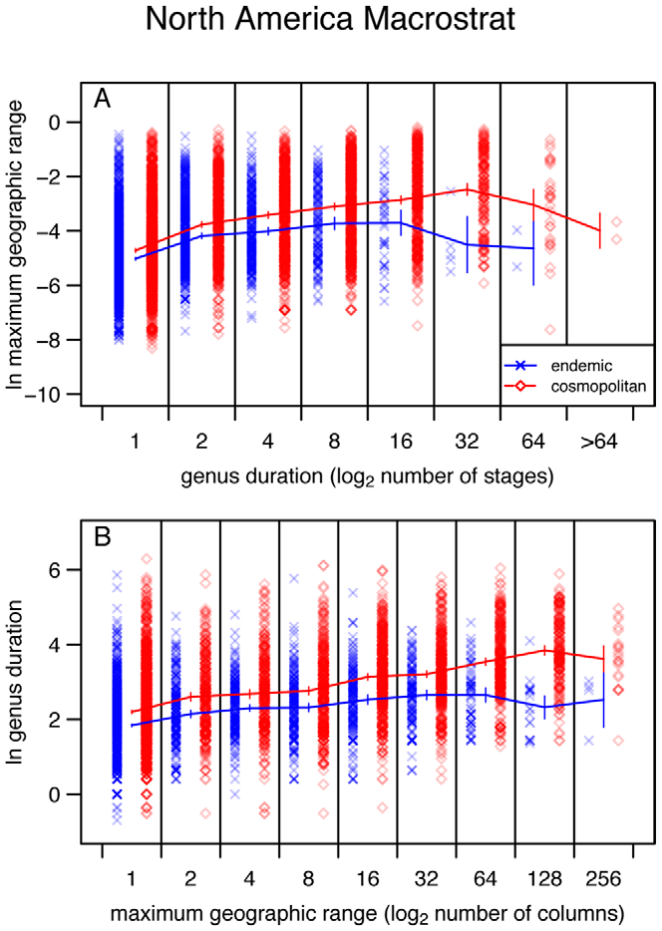
Variation in geographic range and genus duration. (A) Each genus is placed into a log_2_ category based on the number of geologic stages between its first and last appearances, inclusive. For each duration class, the maximum single-stage geographic ranges are plotted for endemic genera (blue crosses) and cosmopolitan genera (red diamonds). The lines show mean values ± two standard errors for endemic and cosmopolitan genera in each duration class. (B) Each genus is placed into a log_2_ category based on the maximum number of geologic summary regions included within its geographic area (dark polygons of Fig. 1). This categorization sorts genera by the maximum single-stage geographic range attained during their histories. For each geographic range class, the stratigraphic durations measured in millions of years are plotted for endemic genera (blue crosses) and cosmopolitan genera (red diamonds). The lines show mean values ± two standard errors for endemic and cosmopolitan genera in each geographic range class. In all plots, symbol outlines are transparent so overlapping points appear darker.

On average, we find that cosmopolitan marine genera from a wide range of Linnaean classes have longer durations within North America (Figs. 2A, S1) and Europe (Figs. 3A, S1) than do endemic genera. Cosmopolitan genera also have wider geographic ranges within North America (Figs. 2B–C, S1) and Europe (Figs. 3B, S1) than endemic genera, and this relationship persists on a per-interval basis throughout most of the Phanerozoic (Fig. 4). Remarkably, cosmopolitan genera exhibit larger geographic ranges within North America and Europe than endemics even when the comparison is restricted to genus duration classes (Figs. 5A). The difference between cosmopolitan and endemic genera is also maintained when geographic range is held constant; cosmopolitan genera have longer temporal durations in North America than do endemics that have comparable geographic ranges (Fig. 5B). The pattern of greater range and duration for cosmopolitan genera is not due to differences in sampling intensity; the relationship persists when the total number of PaleoDB occurrences used to tabulate duration and geographic range is held constant (Fig. S2).

The positive correlation between geographic range and duration is robust and characteristic of a wide range of Linnaean classes, but we are still left with the question of why cosmopolitan genera have larger geographic ranges and persist longer within North America than endemics. There are two hypotheses: 1) Cosmopolitan and endemic genera have similar macroevolutionary dynamics and ecological properties, but frequent immigration events from outside the focal geographic region promote wider within-region geographic spread and longer duration (i.e., the dispersal buffering hypothesis), and 2) cosmopolitan genera have wider regional environmental tolerances and are, therefore, more resistant to local environmental perturbations and disperse more widely than endemic genera (i.e., the niche-breadth hypothesis).

According to the dispersal buffering hypothesis, endemic and cosmopolitan genera are equivalent ecologically, but cosmopolitan genera continuously invade the focal region, which extends their regional durations and geographic spread. Such an assumption of evolutionary and ecological equivalence is typical of many neutral paleontological and neontological biogeographic models [26], [34]–[37]. If the local extinction and reintroduction of cosmopolitan genera occur on a timescale that is resolvable at the stage-level (mean stage duration = 6.2 Myr), then cosmopolitan genera should have, on average, a lower probability of recovery from the fossil record. This is because an absence in a time interval is determined by the sum of the probability of sampling failure plus the probability of actually being absent from the region during that time; whereas the probability of an absence of an endemic genus is related only to the probability of sampling failure (see Materials and Methods). Our data do not support the dispersal buffering hypothesis; the per-interval recovery probabilities of cosmopolitan genera are consistently higher than endemics (Appendix S1; Fig. S3). Moreover, when we compare sampling probabilities within individual paleoenvironmental zones arranged in an onshore-offshore transect (Fig. S4), we find that endemic genera actually have higher probability of sampling than cosmopolitan genera (Fig. S5). Taken together these results show that the higher overall sampling probability of cosmopolitan genera is likely explained by their greater environmental occupancy and not higher rates of dispersal. Insofar as local extinction and immigration can be resolved at the stage-level, these results provide *prima facie* evidence for a very limited causal relationship between dispersal from remote source populations and longevity in North America. This result, of course, does not falsify the buffering hypothesis, but rather represents our best evaluation of the hypothesis in the absence of inter-oceanic basin dispersal rates of marine genera over the Phanerozoic. This result is also inconsistent with the hypothesis that neutral dynamics are operating on the geographic ranges of marine genera over evolutionary time scales.

**Figure 6:**
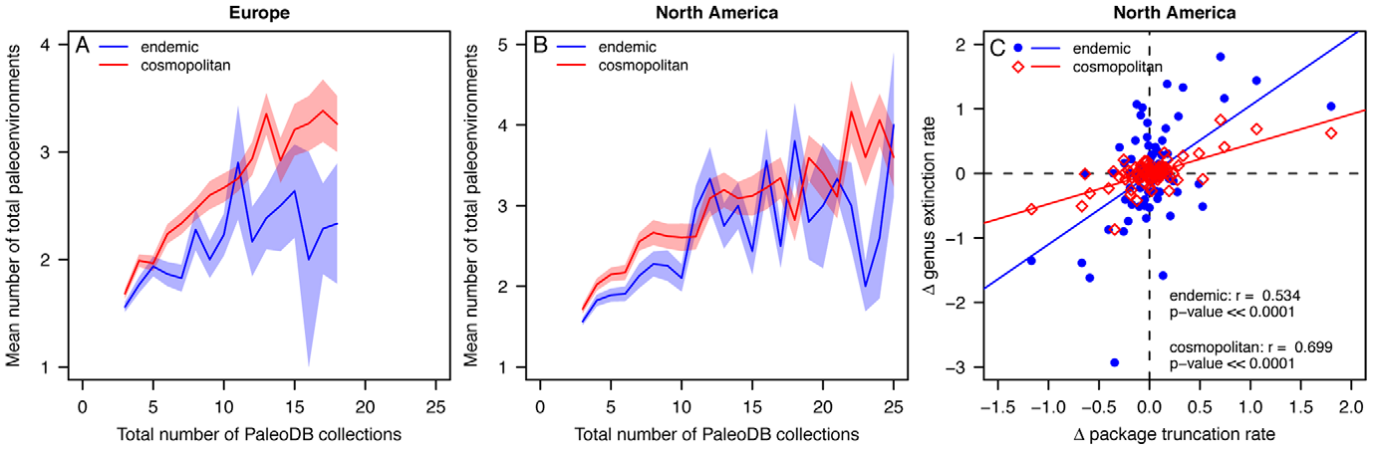
Relationships between paleoenvironments and marine genera. (A) The relationship between the number of PaleoDB collections in Europe in which a genus occurs and the mean number of paleoenvironments occupied by endemic (blue line) and cosmopolitan (red line) genera. Shaded fields are ± one standard error. Only collections that have a paleoenvironment listed in the PaleoDB are included (see Materials and Methods). (B) The relationship between the mean number of paleoenvironments occupied by marine genera in North America and the number of PaleoDB collections. (C) Cross plot of first differences in per-interval, per-capita genus extinction rates vs. sediment package truncation rates (see Materials and Methods) for endemic genera (blue dots) and cosmopolitan genera (red diamonds) in North America. Sloped lines show linear regressions. The slopes of the endemic and cosmopolitan regression lines are 0.88±0.33 and 0.25±0.08, respectively. Note that the two slopes have non-overlapping 95% confidence intervals. Pearson's product-moment correlation coefficients (r) and p-values are given in the bottom right quadrant of the plot.

The alternative to the buffering hypothesis, the niche-breadth hypothesis [2], explains longevity and geographic range as emergent properties of taxa that are able to occupy more habitat types and therefore larger areas within geographic regions. To test the niche-breadth hypothesis, we compared the size of each per interval geographic range to the number of paleoenvironments occupied. Partial correlations were used to determine the relationship between geographic range and environmental breadth independent of sampling effort. Not surprisingly, the partial correlations reduced the strength of the relationship between geographic range size and environmental breadth. However, the relationship is still positive and significant (α = 0.01; Table 1). These results support the niche-breadth hypothesis [2] in that genera occupying larger areas also occupy a larger range of paleoenvironments. Our hypothesis that cosmopolitan genera have wider environmental breadth is also supported by a stronger positive relationship between area and environments than is observed for endemics. Interestingly, calculating geographic range as the proportion of total available marine sedimentary cover occupied by a genus in North America shows a stronger area-environment relationship than the convex hull method. This difference is likely due to the fact that proportional range accounts for variability in geographic range that is driven by changes in actual marine shelf area over time. Furthermore, when the total number of paleoenvironments occupied by a genus during its known evolutionary history are tallied, cosmopolitan genera occupy a greater number of paleoenvironments than their endemic counterparts, even after controlling for sampling intensity (Fig. 6A–B). Although the paleoenvironmental breadth tabulations are based on reduced sample sizes due to missing paleoenvironment data in the PaleoDB, these results suggests that the variety of paleoenvironments or niches occupied by a genus during one time interval (Table 1) and the ability to occupy new environments when conditions change (Fig. 6A–B) both promote survivorship.

**Table 1:** Correlation between per-interval, per-genus geographic range and environmental breadth.

	North America Macrostrat		North America PaleoDB		Europe	
Genus Grouping	r(A,E)	r(A,E)•S	r(A,E)	r(A,E)•S	r(A,E)	r(A,E)•S
all genera	0.49	0.37	0.32	0.26	0.21	0.14
endemic	0.34	0.20	0.17	0.12	0.15	0.10
cosmopolitan	0.53	0.41	0.34	0.29	0.22	0.15

r(A,E) = Pearson product moment correlation coefficients for raw geographic range (A) and number of environments (E). r(A,E)•S = Pearson product moment correlation coefficients for the detrended data. The detrended data are the residuals of linear regressions performed on area vs. number of collections and number of environments vs. number of collections. North America Macrostrat = geographic ranges in North America calculated using Macrostrat data. North America PaleoDB = geographic ranges in North America calculated using only PaleoDB collection locations. Europe = geographic ranges in Europe calculated using only PaleoDB collection locations. All p-values are less than 0.001.

Per interval, per capita rates of genus extinction for endemic and cosmopolitan genera also indicate differences in their sensitivity to regional environmental perturbations. There is a significant positive correlation between changes in rates of marine habitat loss and changes in genus extinction rates for both endemic and cosmopolitan genera in North America (Fig. 6C; see Materials and Methods), but the slope of the relationship is more than a factor of two greater for endemic genera than for cosmopolitan genera. Thus, contractions and rapid shifts in the locations of marine shelf environments in North America affected endemic genera to a greater degree than their cosmopolitan counterparts. The extinction rate result is also consistent with the hypothesis that flexibility in environmental occupancy is an important factor in determining the age-and-area effect.

## Discussion

The geographic range of a genus is a complex emergent property that results from many interacting biological, environmental and macroevolutionary factors [3], [4], [14], [22]. However, determining which, if any, of these factors are important is a non-trivial task. Environmental or niche breadth is one property that likely holds explanatory power. Our results show that endemic marine genera in North America had higher extinction susceptibilities in the face of environmental perturbations (Fig. 6C), which is consistent with the results of Jansson [38], who found that endemic terrestrial species were more likely to survive small amplitude fluctuations in climate than larger ones.

Recent efforts to model Phanerozoic extinction dynamics have been calibrated on the basis of empirical patterns in subsets of taxa, including endemics, which are presumed to be representative of all taxa [36]–[38]. However, our results identify two groups of ecologically and evolutionary diverse genera that consistently display fundamentally different spatial and temporal distributions and sensitivities to environmental perturbations within the same geographic region. The explicit consideration of the differences between endemic and cosmopolitan taxa should help to improve the accuracy of extinction dynamics models. For example, Payne and Finnegan [39] modeled global geographic range extinction selectivity by calibrating the extinction rates of each paleocontinent in each time interval using only endemic genera. Their results show that observed geographic range selectivity is often less than expected, which they attribute to a violation of the neutral assumption of equal extinction probabilities. They propose that differences in physiology among taxa may account for the differences in within-continent extinction probability. Although our results cannot speak to physiology directly, our findings suggest that difference in environmental breadth between endemic and cosmopolitan genera may be one explanation. Of course physiology plays an important role in the distribution of taxa across geochemical and temperature gradients, so habitat breadth and physiology are not necessarily independent. In any case, our results suggest that the initial assumption of equal local extinction susceptibility among genera inhabiting the same paleocontinent may account for much of the lower than expected geographic range selectivity [39].

One of the most striking aspects of our results is that endemic genera respond more strongly to environmental change than do cosmopolitan genera within the same region (Fig. 6C). Lower extinction sensitivity, larger geographic range, and greater survivorship of cosmopolitan genera are likely controlled by greater within-region environmental breadth, not global distribution. Environmental breadth within a region is, therefore, likely to be an important mechanism for overcoming the physical barriers to dispersal, such as those that typically mark boundaries between biogeographic provinces [37], [40], [41]. Our results also raise several important questions that require further investigation. One of these is whether our results apply equally well to endemic and cosmopolitan species or whether they emerge simply as a result of a systematic difference in the number of species within genera. Although this question has in part been answered with living bivalves [22], [40], [42], [43], a direct test of our hypothesis is needed with more taxa and more time intervals. Another interesting line of inquiry concerns the relationships between abundance and geographic range [2]–[4]. Brown's original niche-breadth hypothesis [2] was proposed to explain the spatial distribution of abundances. As more paleontological data on the relative abundances of taxa are collected, a more fully developed test of the niche-breadth hypothesis should be possible.

## Materials and Methods

### Paleontologic and Geologic Data

The paleontological data accessed from the Paleobiology Database (PaleoDB) on 1 February 2011 include 127318 genus occurrences from 7771 marine genera in Canada and the continental U.S.A. (Appendix S2) and 101999 genus occurrences from 9359 marine genera in Europe (Appendix S3). For the purposes of these analyses we included with Europe all of Turkey and excluded Iceland and Svalbard (Fig. 1A). Synonymies and reidentifications were applied to all occurrences based on the taxonomic authority information in the PaleoDB, and only fossil occurrences resolved at the genus level were included (subgenera were not elevated to the genus level). Extant genera were excluded from all analyses to avoid underestimating their stratigraphic durations.

The geologic data in Macrostrat derive principally from stratigraphic summary charts published for Canada by the Geological Survey of Canada [44] and the United States by the American Association of Petroleum Geologists [45], [46]. See Peters and Heim [47] for a full description of the Macrostrat database. The data set consists of 3939 hiatus-bound sedimentary marine packages distributed among 815 geographic regions in Canada and the United States. Hiatus-bound packages are local stratigraphic intervals that record continuous marine deposition at a given temporal resolution, in this case geologic stages, and are bound above and below by stratigraphic hiatuses or intervals of non-marine deposition. See Peters [31] for a full description of the package recognition procedure. Because these packages are hiatus-bound, a first appearance datum (FAD) and last appearance datum (LAD) can be defined for each, allowing environmental turnover to be measured.

### Genus Durations

Marine genus durations were determined using the collection age information in the PaleoDB. In order to maintain consistency between the time scale used here (Appendix S4) and the multiple time scales of the PaleoDB, correlations were established between 389 time intervals in the PaleoDB and 80 time intervals used by Macrostrat [32]. For the purposes of tabulating the longevity of all genera in North America or Europe, including cosmopolitan genera, only occurrences within the focal continents were considered in identifying the FAD and LAD. Furthermore, all analyses of North America and Europe were conducted separately, so genera that occur in both North America and Europe have different durations for each continent.

### Global Genus Distributions

Marine genera with occurrences from North America or Europe were placed into four categories based on the temporal relationship between their PaleoDB occurrences within and outside of the focal region: endemic, immigrant, emigrant and cosmopolitan (Appendix S1). Endemic genera occur in exactly one of the focal regions. Immigrants are those genera whose occurrences in the focal region are all younger than or equal in age to their global last appearance. Emigrants are those genera whose occurrences in the focal region are all older than or equal in age to their global first appearance. Cosmopolitan genera are those with overlapping focal region and non-focal region stratigraphic ranges. Note that PaleoDB occurrences from outside the focal region were only used to place genera into endemism categories. Only endemic and cosmopolitan genera were considered because immigrants and emigrants were indistinguishable from endemic genera in their ranges and durations (Figs. S6, S8, S9). Moreover, including the immigrants and emigrants with the cosmopolitan genera makes no qualitative difference in the results (Fig. S10).

A potential problem with categorizing data based on endemism is that we may be falsely classifying cosmopolitan genera as endemic because occurrences outside of the focal region have not been entered into the PaleoDB. This is expected to be random and should only add noise to our data. To qualitatively estimate the potential magnitude of this type of error we counted the number of days each non-endemic genus resided in the PaleoDB between its first entry and the first entry outside each focal region. The resulting distribution was then compared to the distribution of the residence times in the PaleoDB of currently classified endemic genera (Fig. S12). Most non-endemic genera in both Europe and North America were recognized in the PaleoDB as non-endemic within about 500 days. At the same time, most currently recognized endemic genera have resided in the PaleoDB for more than 1000 days. This result indicates that the potential error added by falsely categorizing endemic genera is likely to be small.

### Geographic Ranges

Geographic range is calculated for each genus in each time interval in which it occurs in R [48] using the *mapproj* [49] and *sp* [50] packages. Genera that occur in multiple time intervals have multiple geographic range estimates. Because the geographic range of a genus during one interval is partially dependent upon its geographic range in the previous time interval, the geographic range estimates for a single genus are not independent [51]. All analyses of geographic range use a single value for each genus, the maximum. In actuality, there is no difference in any of our results if geographic range is treated as statistically independent in each time interval.

Because geographic range is a complex property of species and higher level taxa, its measurement is not straightforward. In these analyses we employ two methods for quantifying the geographic range of fossil marine genera. The first and more conventional method takes the geographic range as the area of the convex hull in square kilometers drawn around all known occurrences for a given taxon in a time interval (Fig. 1A) [6], [8], [52], [53]. The advantage of this method is that it is simple to calculate and only requires age and location information for fossil collections. The disadvantages of this method are that it requires occurrences from at least three distinct localities, thus excluding many rare taxa, and because the analysis only requires knowledge of age and location, there is no context for evaluating the total geologic record available for sampling. The second method for calculating geographic range superimposes fossil occurrences on the known extent of marine sedimentary rocks. Geographic range is calculated as the proportion of the occupied area to the total available area in each time interval. The total available area is defined as the sum of Macrostrat column areas in which there are preserved marine sediments of a given age (all shaded polygons of Fig. 1B). The occupied area is defined by the location of genus occurrences in each time interval. Each PaleoDB occurrence is matched to the closest column within 300 km that contains marine sedimentary rocks of the appropriate age, and then a convex hull is drawn around the polygons that define each of the matched columns. The occupied area is calculated as the sum of the column areas whose centers fall within the convex hull, and the geographic range is the ratio of occupied to total area (Fig. 1B). Note that areas within the convex hull that do not have marine sedimentary rocks of the appropriate age are not included. Calculating geographic range as a proportion of available area accounts for the temporal variation in the areal extent of sedimentary rocks preserved in the geologic record and real changes in marine shelf area [31], [32], [47]. Additionally, Macrostrat allows the geographic ranges of taxa found in only one location to be evaluated by assuming that the time integrated geographic range of a PaleoDB collection is equal to the local extent of a lithostratigraphic unit (shaded polygons of Fig. 1B). This assumption is supported by a time averaging study of modern molluscan assemblages that has shown time averaging within a sedimentary deposit captures a more regional than local diversity-abundance signal [54]. A disadvantage of both methods of geographic range calculation is that they do not explicitly consider discontinuities in the distributions of taxa, and range discontinuities are potentially important when carrying out analyses at the genus level because a single genus could have multiple species with non-overlapping geographic ranges.

Although it is not clear which method is better, knowing the geographic range relative to the maximum possible range is informative. A comparison of the geographic ranges in North America that were calculated using both methods shows good agreement between the two (Spearman rank-order correlation = 0.889; Fig. S11A). A comparison of the raw ranges (results of both methods are in units of km^2^) shows that relative to the convex hull method, the Macrostrat method overestimates small geographic ranges (the lower limit is the area of smallest geologic column) and underestimates the area of large ranges. The Macrostrat method underestimates large ranges relative to the convex hull because it explicitly considers spatial gaps within the geographic range where sampling is impossible because there are no appropriately aged marine sedimentary rocks (Fig. 1B). However, we have identified from this comparison the scale at which spatial patchiness in the geologic record begins to influence observed geographic ranges, which is approximately one-million square kilometers (Fig. S11B). Although future research is needed to understand both the sampling and biological implications of a spatially heterogenous stratigraphic record, the methodology of computing geographic ranges as the proportion of available area occupied by a genus produces qualitatively similar geographic ranges as the traditional convex hull method (Fig. S11A).

### Paleoenvironmental Breadth

PaleoDB paleoenvironments were used to test for differences in the number of habitats occupied by endemic and cosmopolitan genera (Fig. 6A–B). Because not all PaleoDB collections have paleoenvironment information, Figure 6A–B was constructed using a reduced data set. Each paleoenvironment was placed into one of six environmental zones arrayed along an onshore-offshore transect [7], [55] or reefs (Fig. S4). For each genus the total number of distinct paleoenvironment types was tabulated on both a per-interval, per-genus basis (Table 1) and the time-integrated range (Fig. 6A–B). To account for variable sampling intensity, genera sampled from equal numbers of PaleoDB collections were compared. Only genera sampled from 3 to 24 collections are shown (Fig. 6A–B) because endemic genera sampled form greater than 24 collections are rare (but see Fig. S2).

### Macroevolution and macrostratigraphy rates

Because both sedimentary packages and genera have temporal durations defined by FADs and LADs, it is possible to calculate analogous turnover rates for both entities. Rates were calculated following the methodology of Foote [56]. For packages, we calculated marine sediment truncation rates, which are area-weighted measures of the reduction in marine sediment cover through a combination of non-deposition and erosion. To test the hypothesis that genus extinction rates are linked to macrostratigraphic rates of marine sediment truncation, Pearson product moment correlation coefficients (r) were computed on the first differences in analogous extinction metrics for packages and genera. First differences were used in the comparisons because they emphasize interval-to-interval changes, which are important for making causal inferences (Fig. 6C). Finally, linear regressions were used to test the magnitude of the effect of sediment truncation on genus extinction rates.

## Supporting Information

Figure S1 **Key to Linnaean classes plotted in** **Figures 2** **&** **3** **.** (A) Class key to Figure 2A. (B) Class key to Figure 2B. (C) Class key to Figure 2C. (D) Class key to Figure 3A. (E) Class key to Figure 3B.
(TIF)

Figure S2 **Genus durations and ranges controlling for number of occurrences.** (A) Comparison of mean endemic geographic ranges and mean cosmopolitan geographic ranges in Europe based on the convex hull drawn around PaleoDB collections, controlling for the number of occurrences that define each genus' duration. The plotted number indicates the number of occurrences defining the constituent genera. The one-to-one line is plotted for reference. (B) Mean endemic vs. mean cosmopolitan geographic for those genera in North America. Geographic range was calculated as the simple convex hull around PaleoDB occurrences. Plotting conventions are the same as in A. (C) Mean endemic vs. mean cosmopolitan geographic for those genera in North America. Geographic range was calculated as the proportion of occupied sedimentary cover. Plotting conventions are the same as in A. (D) Comparison of mean endemic duration and mean cosmopolitan duration in Europe, controlling for the number of occurrences that define each genus' duration. Each number is plotted as the mean of all genera defined by the same number of occurrences. Plotting conventions are the same as in A. (E) Mean endemic vs. mean cosmopolitan durations for those genera in North America. Plotting conventions are the same as in A.
(TIF)

Figure S3 **Sampling probability for endemic and cosmopolitan genera.** Sampling probability is the proportion of time intervals, exclusive of the range ends, that have a sampled occurrence for each genus known to exist during that time interval [S1, S2]. (A) The time series of mean sampling probabilities with one standard error of mean. The time scale abbreviations are the same as in Figure 3A. (B) Box plots of the sampling probabilities of all genera. Notches show 95% confidence intervals for medians (2-sided Wilcox test: W = 2076.5, p-value = 0.0006). (C) Mean sampling probability for endemic and cosmopolitan genera grouped by genus longevity into log_2_ bins. Because the time intervals of the FAD and LADs are not included in the analysis, only genera that span a minimum of three time intervals are included. Error bars are ± two standard errors.
(TIF)

Figure S4 **The marine paleoenvironment categories and their sub-environments used to estimate habitat breadth.** The environments in regular type are the PaleoDB collection environments and the categories used in the habitat breadth analysis (Fig. 6) are in bold-face. The parenthetical numbers correspond to the paleoenvironment categories, arrayed in an onshore-offshore transect, used by Sepkoski [38]. Sepkoski excluded reefs, but they are included here.
(TIF)

Figure S5 **North American sampling probabilities for each paleoenvironment.** Box plots of the sampling probabilities of each endemic and cosmopolitan genus within a particular paleoenvironmental zone. Only the stratigraphic range between the first and last genus occurrence within the zone of consideration is considered for each genus. These plots demonstrate that cosmopolitan genera are also more completely sampled within single environmental zones during the portions of time in which they are observed in those zones.
(TIF)

Figure S6 **Time series of mean geographic ranges.** Comparisons of mean geographic ranges within North America among the three geographic genus categories: endemic (black), immigrant (blue) and emigrant (red). The endemic data and plotting conventions are the same as in Figure 4. (A) North American genera with geographic range calculated as the proportion of available sediments. (B) North American genera with geographic range calculated as the convex hull around PaleoDB collections. (C) European genera with geographic range calculated as the convex hull around PaleoDB collections.
(TIF)

Figure S7 **Mean genus duration for Linnaean classes.** (A) Endemic vs. immigrant duration within North America. (B) Endemic vs. immigrant duration within Europe. (C) Endemic vs. emigrant duration within North America. (D) Endemic vs. emigrant duration within Europe. The red crosses are ± two standard errors around the mean for all genera in each category. Plotting conventions are the same as in Figure 2.
(TIF)

Figure S8 **Mean genus geographic range for Linnaean classes.** (A) Endemic vs. immigrant duration within North America with geographic range calculated as the proportion of available sediments. (B) Endemic vs. immigrant geographic range within North America with geographic range calculated as the convex hull around PaleoDB collections. (C) Endemic vs. immigrant duration within Europe with geographic range calculated as the convex hull around PaleoDB collections. (D) Endemic vs. emigrant duration within North America with geographic range calculated as the proportion of available sediments. (E) Endemic vs. emigrant geographic range within North America with geographic range calculated as the convex hull around PaleoDB collections. (F) Endemic vs. emigrant duration within Europe with geographic range calculated as the convex hull around PaleoDB collections. Plotting conventions are the same as in Figure 2.
(TIF)

Figure S9 **Variation in geographic range and genus duration for endemic, immigrant and emigrant genera.** Note that there are no significant differences among endemic, immigrant and emigrant genera. The endemic data and plotting conventions are the same as in Figure 5.
(TIF)

Figure S10 **Mean genus duration and geographic range for Linnaean classes with all non-endemic genera pooled.** This figure should be compared to Figures 2 and 3. For this figure, cosmopolitan, immigrant and emigrant genera are pooled and compared to endemic genera. Note that pooling all non-endemic genera does not qualitatively change the relationships observed cosmopolitan genera alone. Error bars are ± one standard error of class mean. The one-to-one line (dashed) is plotted for reference. Only one value for each genus, mean geographic range, is used in the per class calculations. The red crosses are ± two standard errors around the mean for all genera in each category. (A) Endemic vs. cosmopolitan duration within North America. (B) Endemic vs. cosmopolitan geographic range, calculated as a convex hull around PaleoDB collections, within North America. (C) Endemic vs. cosmopolitan geographic range, calculated as proportion of the total available rock area occupied by a genus within North America. (D) Endemic vs. cosmopolitan duration within Europe. (E) Endemic vs. cosmopolitan geographic range, calculated as a convex hull around PaleoDB collections, within Europe.
(TIF)

Figure S11 **Comparison of per-interval, per-genus geographic ranges calculated using the PaleoDB and Macrostrat.** (A) Convex hull area vs. the proportion of occupied area. The Spearman rank-order correlation coefficient (ρ) and p-value are shown in the bottom left. (B) Convex hull area vs. the total occupied area as estimated from Macrostrat. The data plotted on the y-axis differ from those in (A) in that they are not divided by the total available rock area. The oblique horizontal line is the one-to-one line and is shown for reference. The dotted lines mark the approximate point where spatial gaps in marine sedimentary cover become important and the Macrostrat method produces smaller geographic areas than the convex hull method (e^13.8^ km^2^). All points are translucent so overlapping points appear darker.
(TIF)

Figure S12 **Residence time of endemic genera and time for recognition of non-endemic genera.** The histogram in the upper panel shows the number of days each genus endemic to North America has been in the PaleoDB. The solid and dashed lines show the mean and middle 50%, respectively, number of days genera that are not endemic to North America took to be recognized as non-endemic. If, for example, the first occurrence entered into the PaleoDB for a globally distributed genus is located in North America, that genus would be recognized in these analyses as endemic until an occurrence from outside North America is entered. The main point of this figure is to show that most endemic genera have been entered into the PaleoDB long enough to be confidently classified as endemic. The lower panel shows the same information for Europe.
(TIF)

Appendix S1 Supplemental methods.
(RTF)

Appendix S2 Data file with FADs, LADs, number of PaleoDB occurrences, number of paleoenvironments occupied, Linnaean hierarchy and status as endemic or cosmopolitan for each genus in North America.
(CSV)

Appendix S3 Data file with FADs, LADs, number of PaleoDB occurrences, number of paleoenvironments occupied, Linnaean hierarchy and status as endemic or cosmopolitan for each genus in Europe.
(CSV)

Appendix S4 Data file with geologic time scale, total area of marine rocks and per interval raw geographic ranges.
(CSV)
