# Supplementary figures and images for: Regional Environmental Breadth Predicts Geographic Range and Longevity in Fossil Marine Genera
Source: PLoS One. 2011 May 4;6(5):e18946. doi: 10.1371/journal.pone.0018946 (PMC3087726; doi:10.1371/journal.pone.0018946)

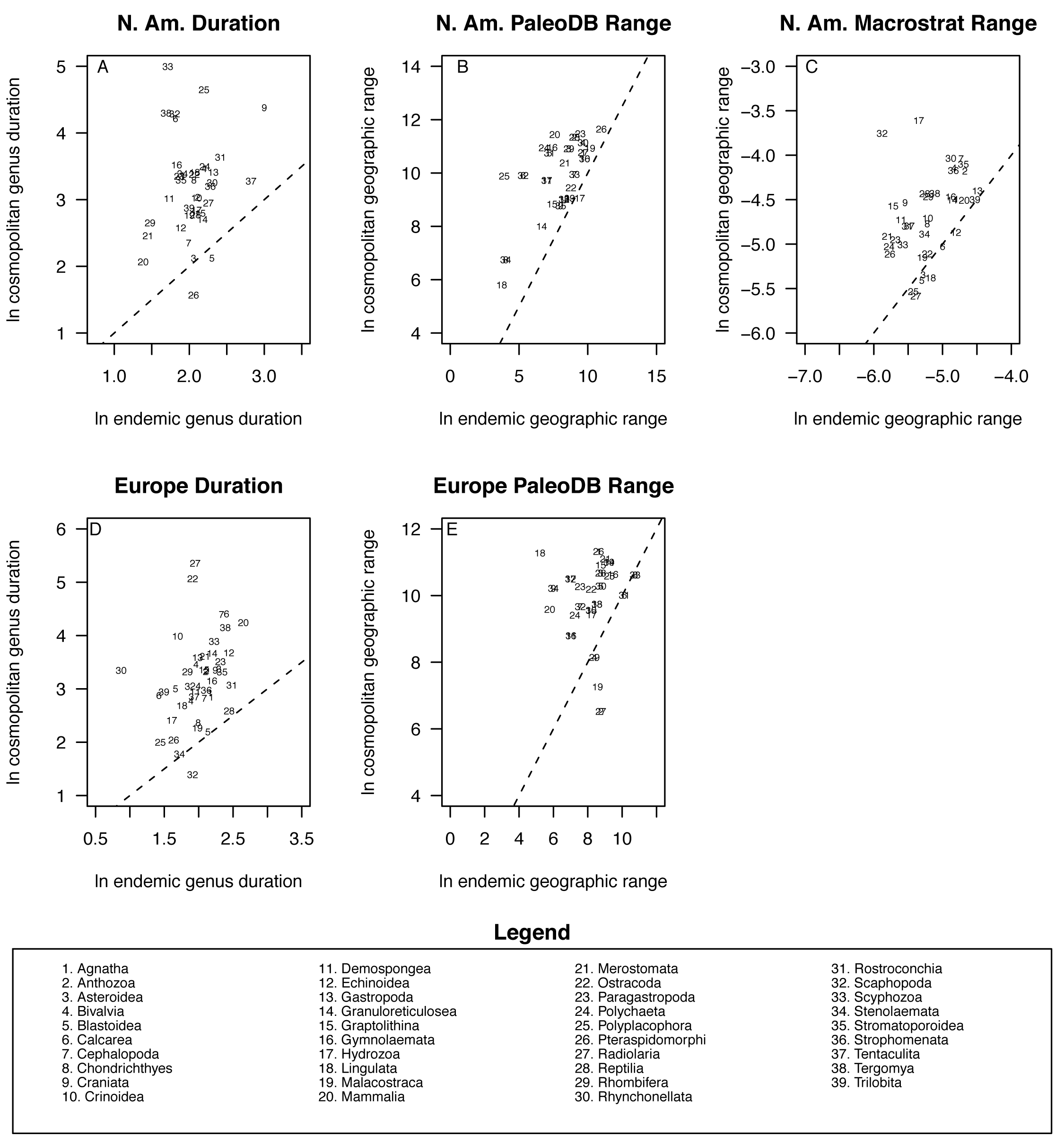

Supplement: Figure S1 — Key to Linnaean classes plotted in Figures 2 & 3 . (A) Class key to Figure 2A. (B) Class key to Figure 2B. (C) Class key to Figure 2C. (D) Class key to Figure 3A. (E) Class key to Figure 3B. (TIF) [file pone.0018946.s001.tif]

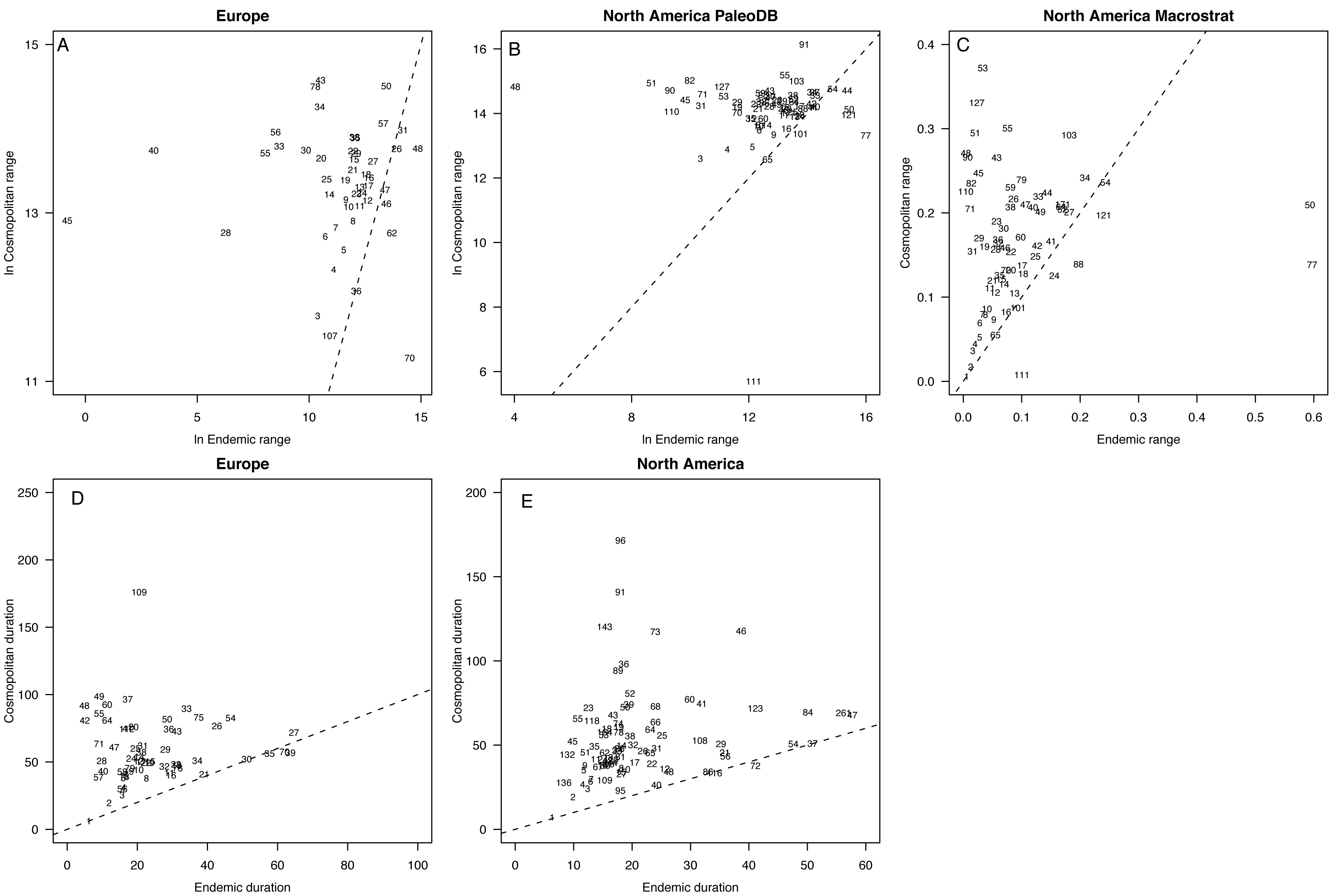

Supplement: Figure S2 — Genus durations and ranges controlling for number of occurrences. (A) Comparison of mean endemic geographic ranges and mean cosmopolitan geographic ranges in Europe based on the convex hull drawn around PaleoDB collections, controlling for the number of occurrences that define each genus' duration. The plotted number indicates the number of occurrences defining the constituent genera. The one-to-one line is plotted for reference. (B) Mean endemic vs. mean cosmopolitan geographic for those genera in North America. Geographic range was calculated as the simple convex hull around PaleoDB occurrences. Plotting conventions are the same as in A. (C) Mean endemic vs. mean cosmopolitan geographic for those genera in North America. Geographic range was calculated as the proportion of occupied sedimentary cover. Plotting conventions are the same as in A. (D) Comparison of mean endemic duration and mean cosmopolitan duration in Europe, controlling for the number of occurrences that define each genus' duration. Each number is plotted as the mean of all genera defined by the same number of occurrences. Plotting conventions are the same as in A. (E) Mean endemic vs. mean cosmopolitan durations for those genera in North America. Plotting conventions are the same as in A. (TIF) [file pone.0018946.s002.tif]

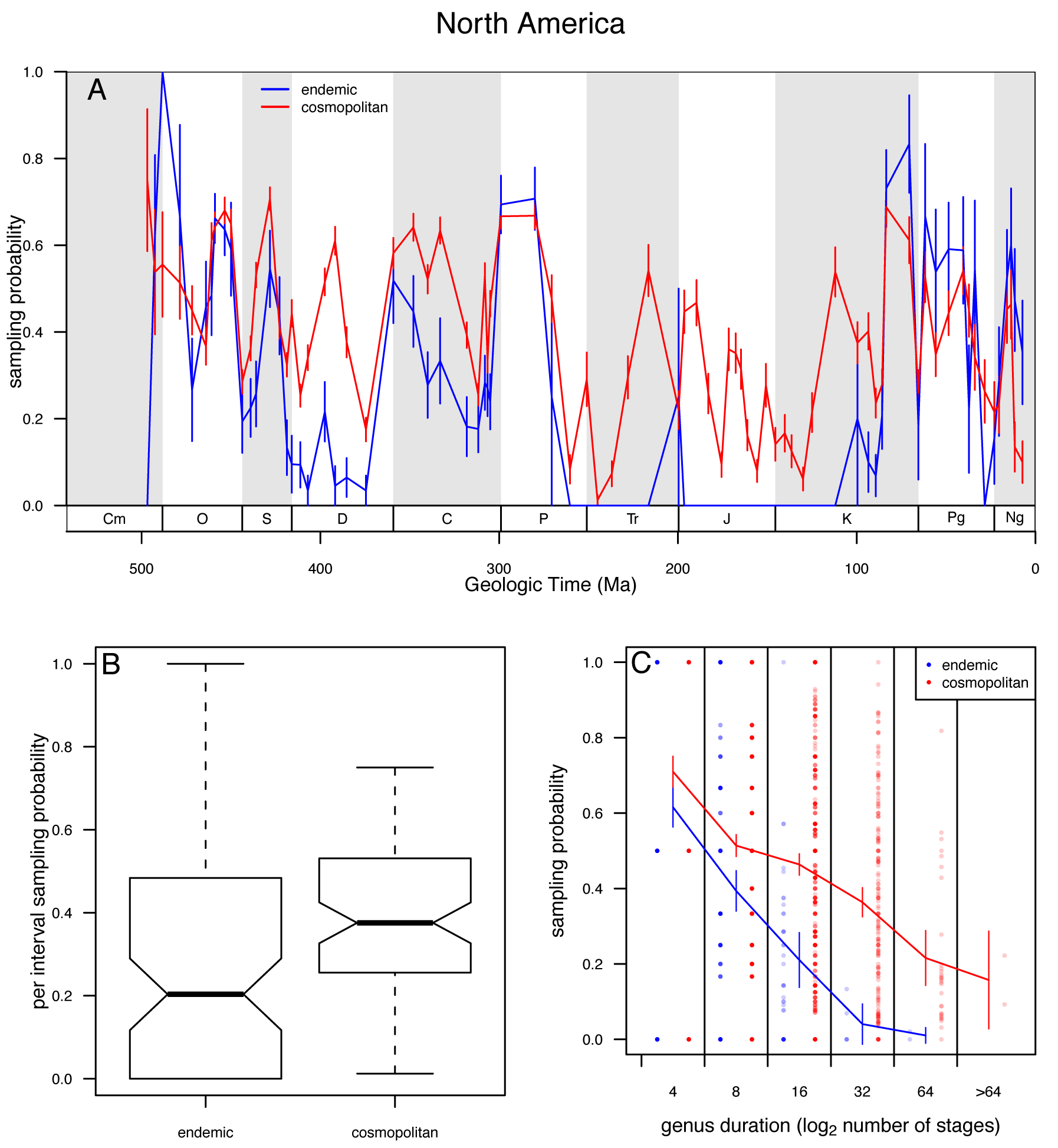

Supplement: Figure S3 — Sampling probability for endemic and cosmopolitan genera. Sampling probability is the proportion of time intervals, exclusive of the range ends, that have a sampled occurrence for each genus known to exist during that time interval [S1, S2]. (A) The time series of mean sampling probabilities with one standard error of mean. The time scale abbreviations are the same as in Figure 3A. (B) Box plots of the sampling probabilities of all genera. Notches show 95% confidence intervals for medians (2-sided Wilcox test: W = 2076.5, p-value = 0.0006). (C) Mean sampling probability for endemic and cosmopolitan genera grouped by genus longevity into log2 bins. Because the time intervals of the FAD and LADs are not included in the analysis, only genera that span a minimum of three time intervals are included. Error bars are ± two standard errors. (TIF) [file pone.0018946.s003.tif]

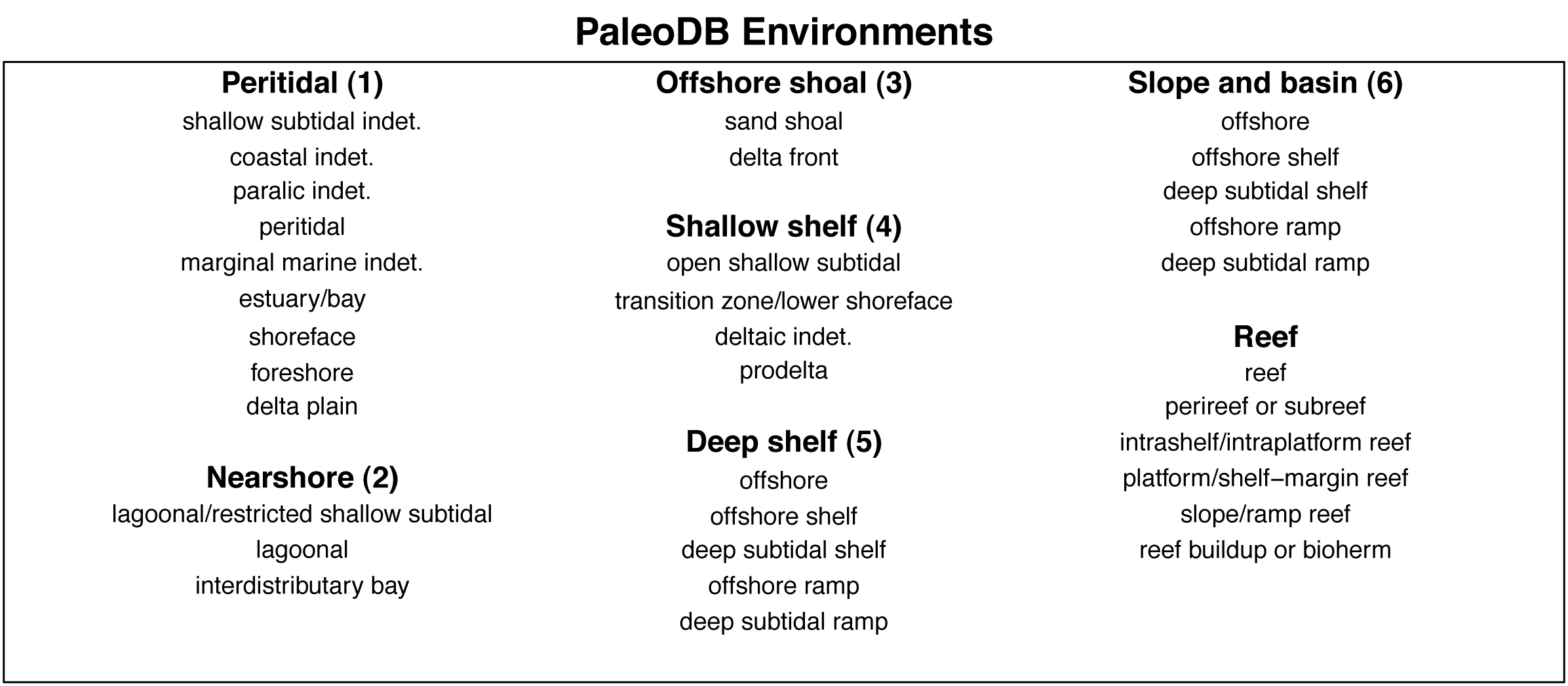

Supplement: Figure S4 — The marine paleoenvironment categories and their sub-environments used to estimate habitat breadth. The environments in regular type are the PaleoDB collection environments and the categories used in the habitat breadth analysis (Fig. 6) are in bold-face. The parenthetical numbers correspond to the paleoenvironment categories, arrayed in an onshore-offshore transect, used by Sepkoski [38]. Sepkoski excluded reefs, but they are included here. (TIF) [file pone.0018946.s004.tif]

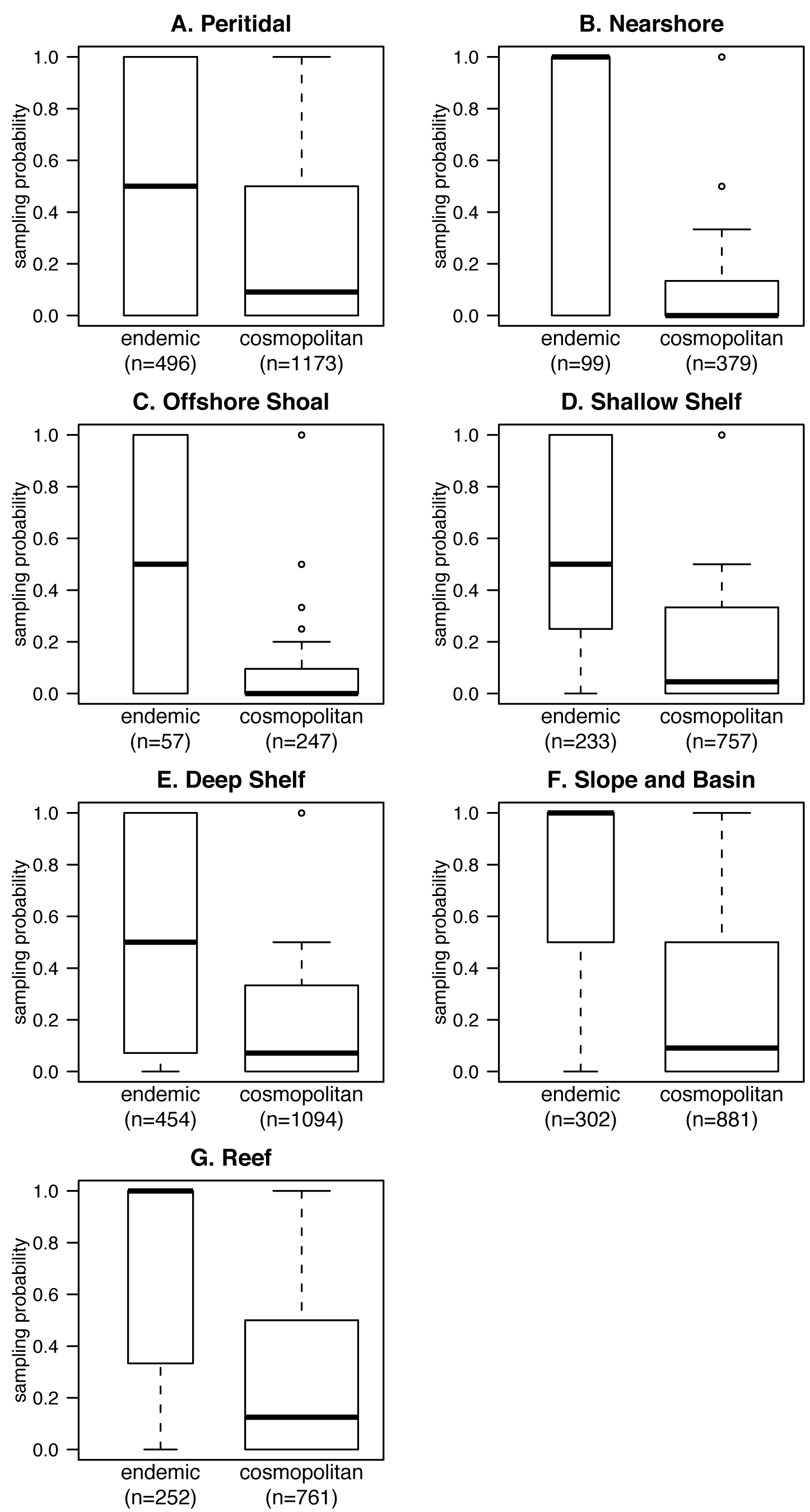

Supplement: Figure S5 — North American sampling probabilities for each paleoenvironment. Box plots of the sampling probabilities of each endemic and cosmopolitan genus within a particular paleoenvironmental zone. Only the stratigraphic range between the first and last genus occurrence within the zone of consideration is considered for each genus. These plots demonstrate that cosmopolitan genera are also more completely sampled within single environmental zones during the portions of time in which they are observed in those zones. (TIF) [file pone.0018946.s005.tif]

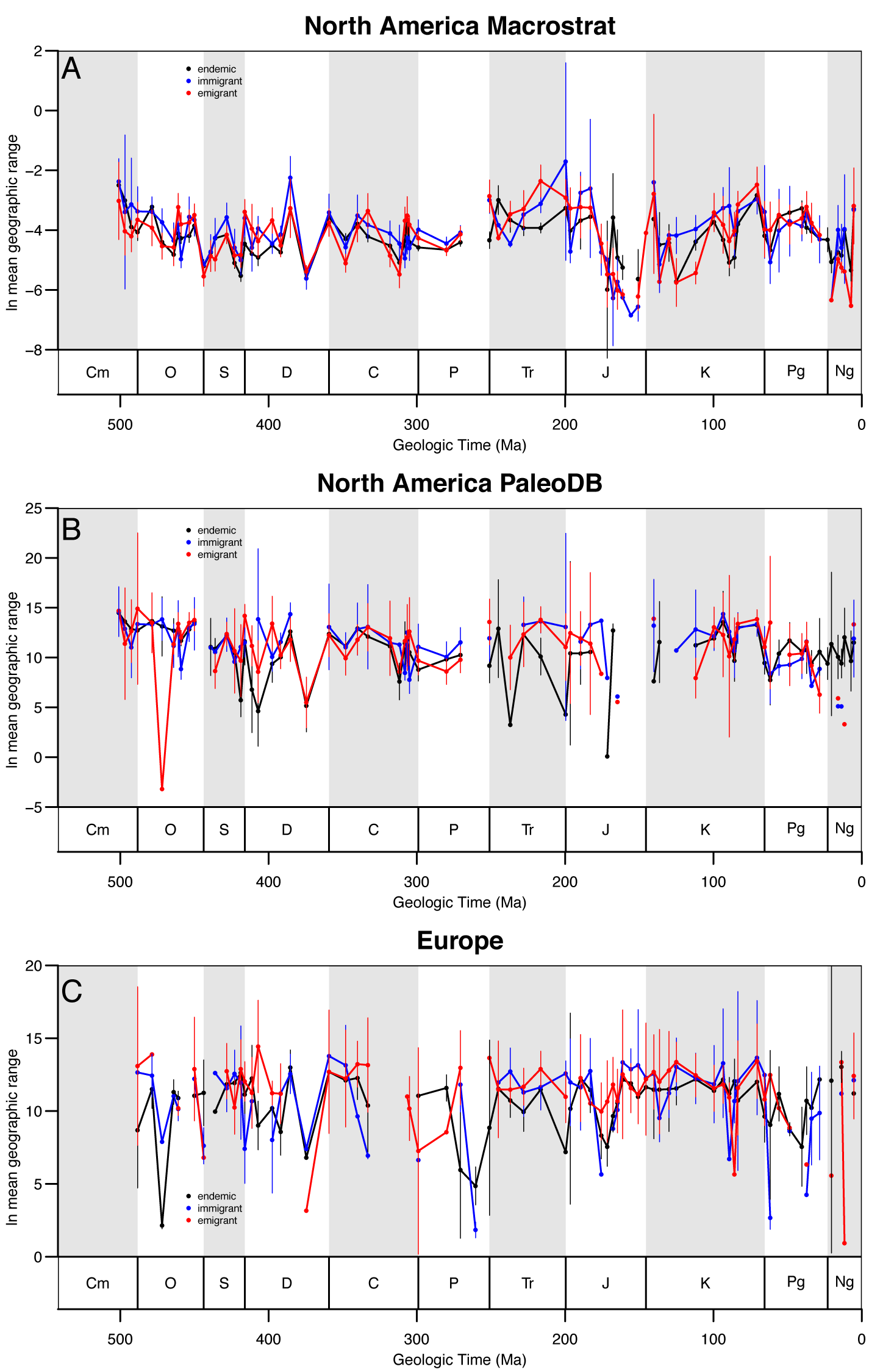

Supplement: Figure S6 — Time series of mean geographic ranges. Comparisons of mean geographic ranges within North America among the three geographic genus categories: endemic (black), immigrant (blue) and emigrant (red). The endemic data and plotting conventions are the same as in Figure 4. (A) North American genera with geographic range calculated as the proportion of available sediments. (B) North American genera with geographic range calculated as the convex hull around PaleoDB collections. (C) European genera with geographic range calculated as the convex hull around PaleoDB collections. (TIF) [file pone.0018946.s006.tif]

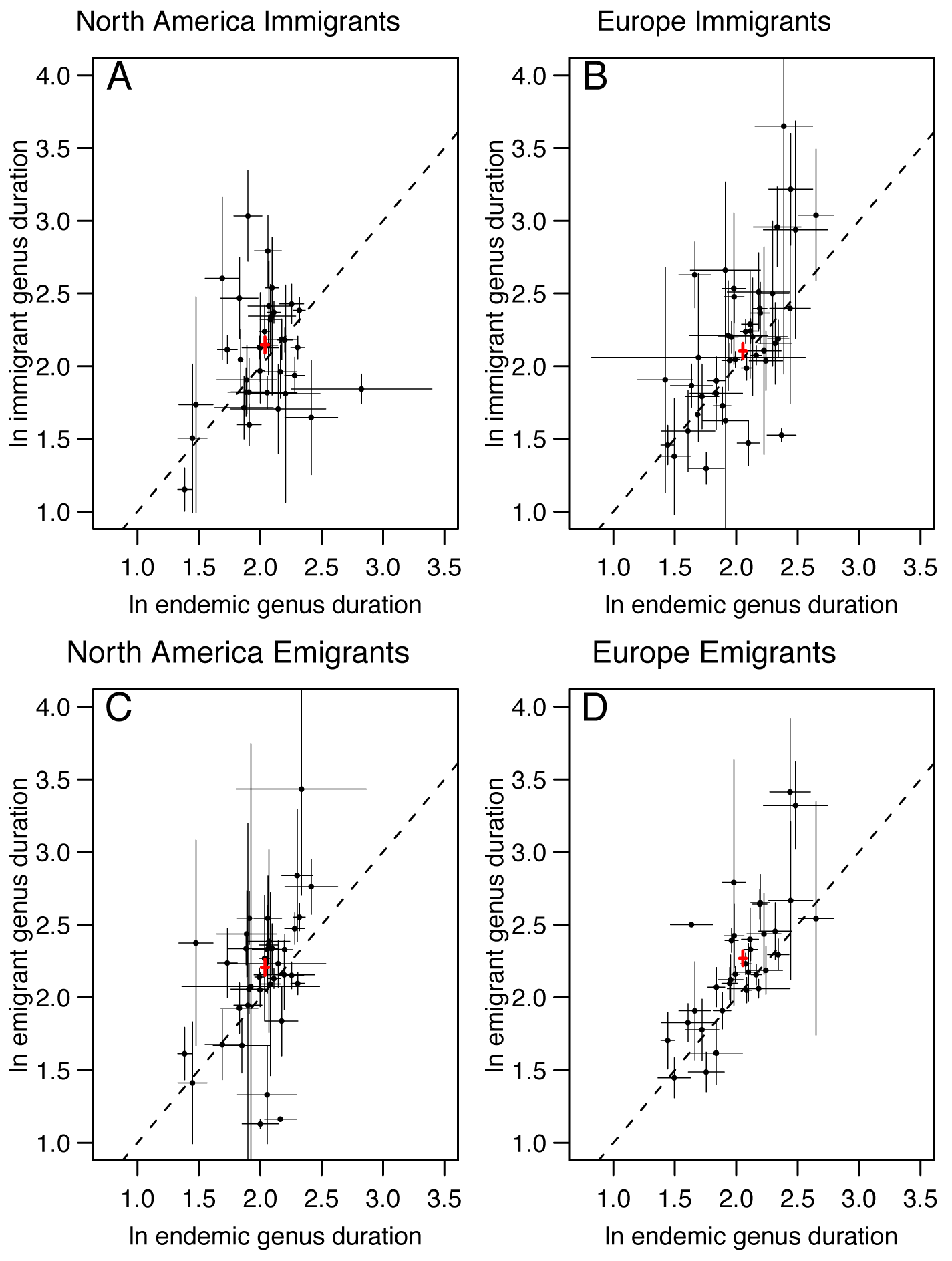

Supplement: Figure S7 — Mean genus duration for Linnaean classes. (A) Endemic vs. immigrant duration within North America. (B) Endemic vs. immigrant duration within Europe. (C) Endemic vs. emigrant duration within North America. (D) Endemic vs. emigrant duration within Europe. The red crosses are ± two standard errors around the mean for all genera in each category. Plotting conventions are the same as in Figure 2. (TIF) [file pone.0018946.s007.tif]

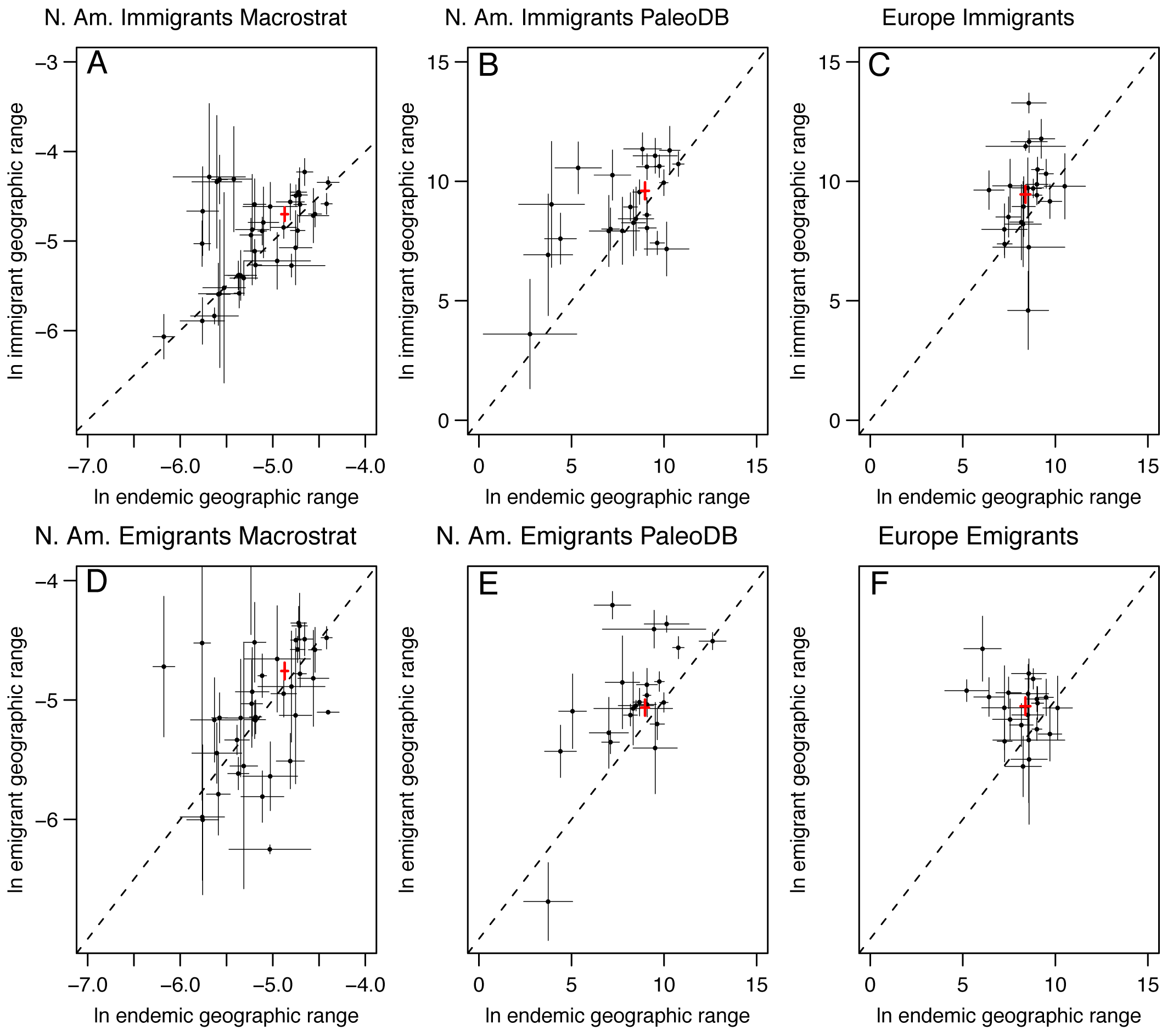

Supplement: Figure S8 — Mean genus geographic range for Linnaean classes. (A) Endemic vs. immigrant duration within North America with geographic range calculated as the proportion of available sediments. (B) Endemic vs. immigrant geographic range within North America with geographic range calculated as the convex hull around PaleoDB collections. (C) Endemic vs. immigrant duration within Europe with geographic range calculated as the convex hull around PaleoDB collections. (D) Endemic vs. emigrant duration within North America with geographic range calculated as the proportion of available sediments. (E) Endemic vs. emigrant geographic range within North America with geographic range calculated as the convex hull around PaleoDB collections. (F) Endemic vs. emigrant duration within Europe with geographic range calculated as the convex hull around PaleoDB collections. Plotting conventions are the same as in Figure 2. (TIF) [file pone.0018946.s008.tif]

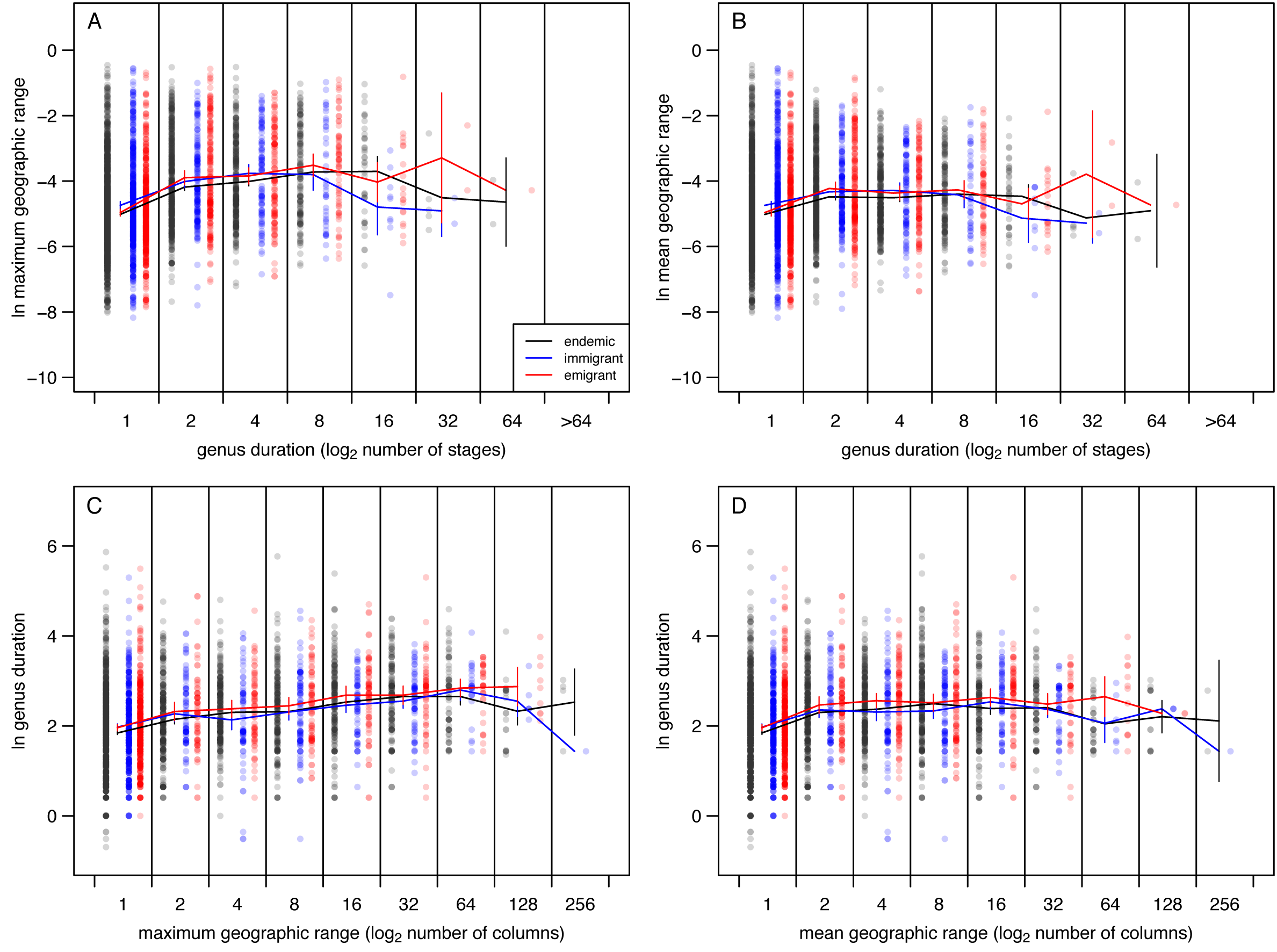

Supplement: Figure S9 — Variation in geographic range and genus duration for endemic, immigrant and emigrant genera. Note that there are no significant differences among endemic, immigrant and emigrant genera. The endemic data and plotting conventions are the same as in Figure 5. (TIF) [file pone.0018946.s009.tif]

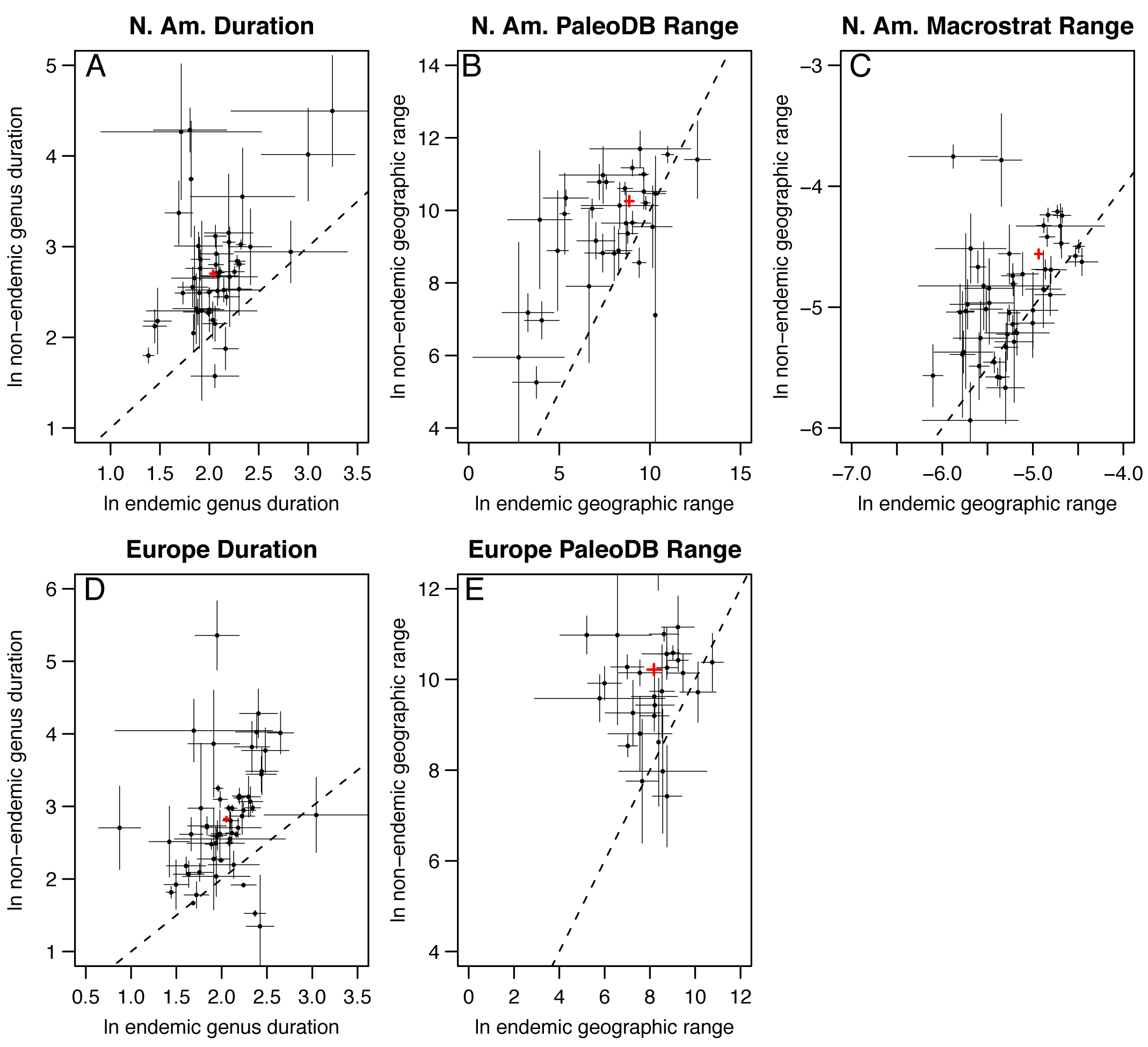

Supplement: Figure S10 — Mean genus duration and geographic range for Linnaean classes with all non-endemic genera pooled. This figure should be compared to Figures 2 and 3. For this figure, cosmopolitan, immigrant and emigrant genera are pooled and compared to endemic genera. Note that pooling all non-endemic genera does not qualitatively change the relationships observed cosmopolitan genera alone. Error bars are ± one standard error of class mean. The one-to-one line (dashed) is plotted for reference. Only one value for each genus, mean geographic range, is used in the per class calculations. The red crosses are ± two standard errors around the mean for all genera in each category. (A) Endemic vs. cosmopolitan duration within North America. (B) Endemic vs. cosmopolitan geographic range, calculated as a convex hull around PaleoDB collections, within North America. (C) Endemic vs. cosmopolitan geographic range, calculated as proportion of the total available rock area occupied by a genus within North America. (D) Endemic vs. cosmopolitan duration within Europe. (E) Endemic vs. cosmopolitan geographic range, calculated as a convex hull around PaleoDB collections, within Europe. (TIF) [file pone.0018946.s010.tif]

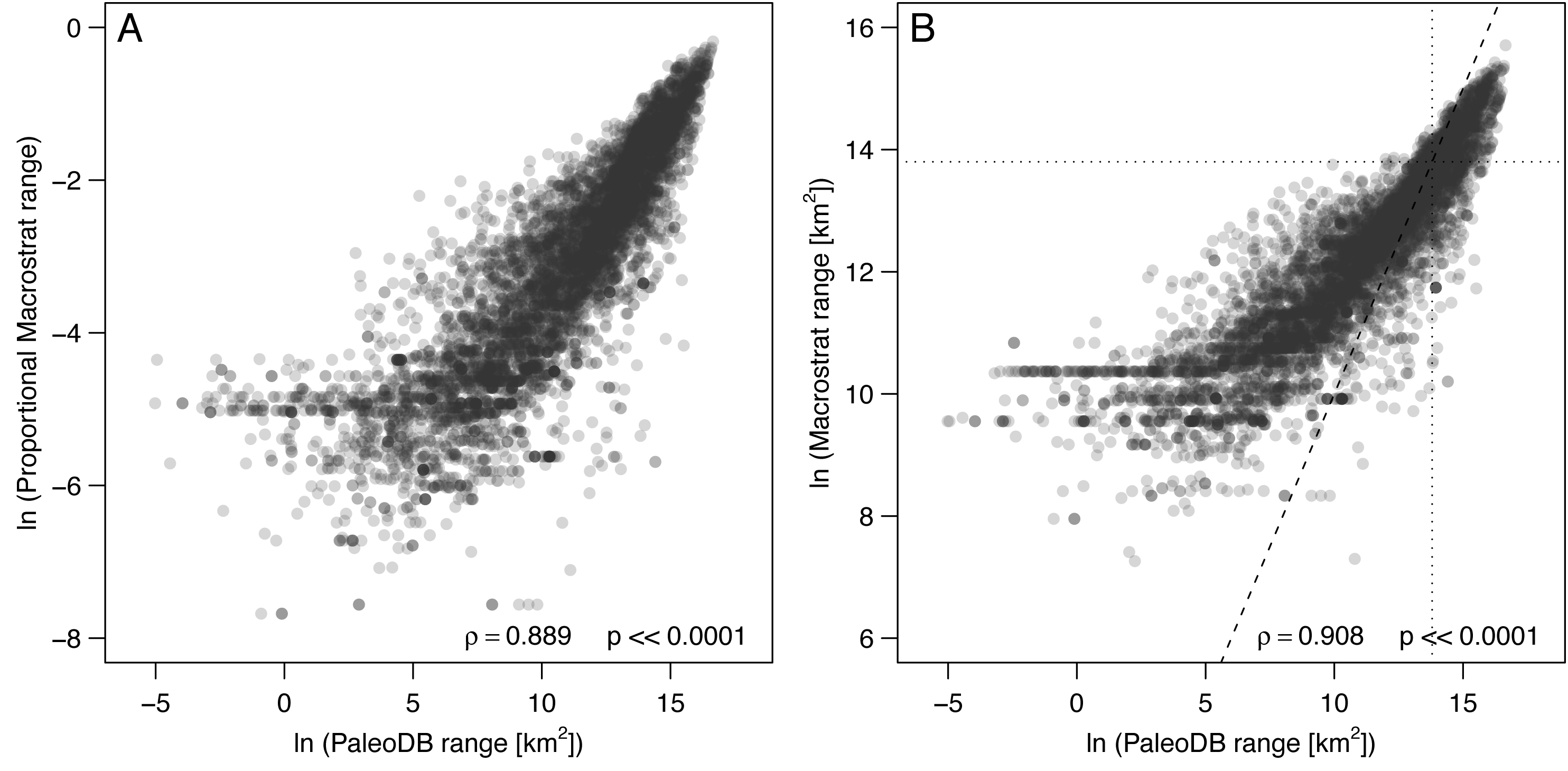

Supplement: Figure S11 — Comparison of per-interval, per-genus geographic ranges calculated using the PaleoDB and Macrostrat. (A) Convex hull area vs. the proportion of occupied area. The Spearman rank-order correlation coefficient (ρ) and p-value are shown in the bottom left. (B) Convex hull area vs. the total occupied area as estimated from Macrostrat. The data plotted on the y-axis differ from those in (A) in that they are not divided by the total available rock area. The oblique horizontal line is the one-to-one line and is shown for reference. The dotted lines mark the approximate point where spatial gaps in marine sedimentary cover become important and the Macrostrat method produces smaller geographic areas than the convex hull method (e13.8 km2). All points are translucent so overlapping points appear darker. (TIF) [file pone.0018946.s011.tif]

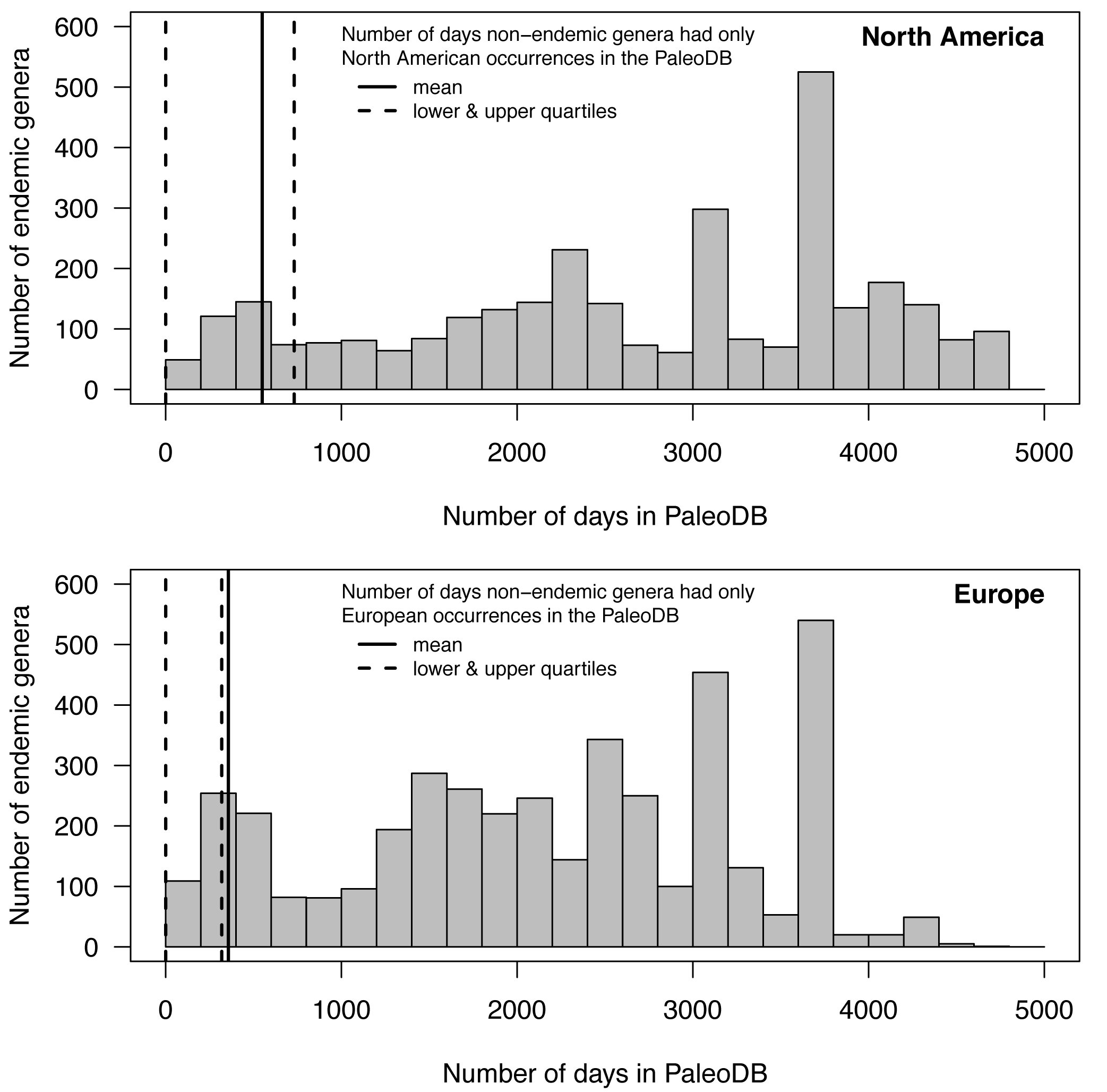

Supplement: Figure S12 — Residence time of endemic genera and time for recognition of non-endemic genera. The histogram in the upper panel shows the number of days each genus endemic to North America has been in the PaleoDB. The solid and dashed lines show the mean and middle 50%, respectively, number of days genera that are not endemic to North America took to be recognized as non-endemic. If, for example, the first occurrence entered into the PaleoDB for a globally distributed genus is located in North America, that genus would be recognized in these analyses as endemic until an occurrence from outside North America is entered. The main point of this figure is to show that most endemic genera have been entered into the PaleoDB long enough to be confidently classified as endemic. The lower panel shows the same information for Europe. (TIF) [file pone.0018946.s012.tif]
