# Supplementary material for: Regional Environmental Breadth Predicts Geographic Range and Longevity in Fossil Marine Genera
Source: PLoS One. 2011 May 4;6(5):e18946. doi: 10.1371/journal.pone.0018946 (PMC3087726; doi:10.1371/journal.pone.0018946)
Supplement: Appendix S1 — Supplemental methods. (RTF) [file pone.0018946.s013.rtf]

Supporting Information For: Local Environmental Breadth Determines Geographic Range and Longevity in Fossil Marine GeneraNoel A. Heim* & Shanan E. Peters*To whom correspondence should be addressed.  E-mail: nheim@wisc.eduEndemics, Immigrants and EmigrantsIn all measures of geographic range, immigrant and emigrant genera were indistinguishable from endemic genera (S6,S7,S9).  In essence immigrants and emigrants have the properties of North American endemic genera when they are indeed endemic to the region.  However, because immigrants and emigrants do occur outside of North America during their evolutionary histories they do not fit our definition of endemism so we excluded them from our analyses.  However, there are no differences in the results when we include immigrants and emigrants with the cosmopolitan genera.  With this more relaxed definition of cosmopolitanism, cosmopolitan genera still persist longer, and have wider geographic ranges, but the magnitude of the difference is somewhat smaller.  The biogeographic dynamics of immigrant and emigrant genera are the topic of a forthcoming manuscript by us.Sampling ProbabilitiesAll tabulations of geographic range, duration and sampling probabilities are based solely on North American occurrences.  This assumption is valid for endemic ranges, but potentially invalid the ranges of cosmopolitan genera within North America because genera could become regionally extinct then reestablished by immigration.  The potential influence of multiple “seedings” of cosmopolitan genera in North America was tested with sampling probabilities [S1, S2].  For endemic genera, sampling failure is the only explanation for unsampled time bins in its geologic duration.  For cosmopolitan genera, however, gaps in stratigraphic durations can be due to either sampling failure, as in endemics, or there can be true gaps where the genus was temporarily absent from North America.  Thus, if cosmopolitan genera frequently experienced temporary absences from North America, it is expected that cosmopolitan genera would have a lower sampling probability.  The sampling probability for each genus is the proportion of time bins between its FAD and LAD, exclusive, in which the genus was actually sampled.  Because the time bins of the FADs and LADs are not counted in the total number of potentially sampled bins for each genus, only genera with a total duration of three or more time bins were included.  Although there is variation, cosmopolitan genera show greater sampling probabilities than endemics through the Phanerozoic (Fig. S3A).  Furthermore, cosmopolitan genera tend to have a higher sampling probability overall (Fig. S3B), and when genera are grouped according to longevity, cosmopolitan genera are more completely sampled except for those with the shortest durations, where endemics and cosmopolitan genera have equivocal sampling probabilities (Fig. S3C).Another potential sampling issues is the false categorization of endemic genera because not enough occurrences have been entered into the PaleoDB.  Random errors such as these are not likely to adversely alter the results [S3, S4], however we have qualitatively estimated the potential magnitude of errors from incorrectly categorizing genera as endemics simply because occurrence from outside North America or Europe have been entered yet (Fig. S12).  We have compared the characteristic time for the recognition of non-endemic genera by counting the number of days between each genus first entry in North America (or Europe) and the first entry outside the focal region.  Genera whose first PaleoDB entry is outside of the focal region are included (zero days).  The mean and middle 50% of the resulting distribution is compared to the number of days currently recognized endemic genera have been listed in the PaleoDB.  Although some of the endemic genera are certainly identified in error, the vast majority of the endemic genera have been known by the PaleoDB long enough that we are confident the number of genera incorrectly categorized as endemic is small.ReferencesS1	Paul, C. R. C. in Problems of Phylogenetic Reconstruction (Systematics Association Special Volume No. 21)   (eds K. A. Joysey & A. E. Friday)  75-117 (Academic Press, 1982).S2	Foote, M. & Raup, D. M. Fossil preservation and the stratigraphic ranges of taxa. Paleobiology 22, 121-140 (1996).S3	Sepkoski JJ, Jr. (1993) Ten years in the library: new data confirm paleontological patterns. Paleobiology 19: 43-51.S4	Adrain JM, Westrop SR (2000) An empirical assessment of taxic paleobiology. Science 289: 110-112.
